# Genome-wide identification of loci modifying spike-branching in tetraploid wheat

**DOI:** 10.1007/s00122-020-03743-5

**Published:** 2021-05-07

**Authors:** Gizaw M. Wolde, Mona Schreiber, Corinna Trautewig, Axel Himmelbach, Shun Sakuma, Martin Mascher, Thorsten Schnurbusch

**Affiliations:** 1 Leibniz Institute of Plant Genetics and Crop Plant Research (IPK), Corrensstr. 3, OT Gatersleben, 06466 Seeland, Germany; 2 Faculty of Natural Sciences III, Institute of Agricultural and Nutritional Sciences, Martin Luther University Halle-Wittenberg, 06120 Halle, Germany; 3 Present Address: Department of Plant Sciences One Shields Avenue, University of California, Davis, CA 95616 USA; 4 Present Address: Faculty of Agriculture, Tottori University, 4-101 Koyama-cho Minami, Tottori, 680-8553 Japan

## Abstract

**Key message:**

Genetic modification of spike architecture is essential for improving wheat yield. Newly identified loci for the ‘Miracle wheat’ phenotype on chromosomes 1AS and 2BS have significant effects on spike traits.

**Abstract:**

The wheat (*Triticum* ssp.) inflorescence, also known as a spike, forms an unbranched inflorescence in which the inflorescence meristem generates axillary spikelet meristems (SMs) destined to become sessile spikelets. Previously, we identified the putatively causative mutation in the *branched head*^*t*^ (*bh*^*t*^) gene (*TtBH*-*A1*) of tetraploid wheat (*T. turgidum* convar. *compositum* (L.f.) Filat.) responsible for the loss of SM identity, converting the non-branching spike to a branched wheat spike. In the current study, we performed whole-genome quantitative trait loci (QTL) analysis using 146 recombinant inbred lines (RILs) derived from a cross between spike-branching wheat (‘Miracle wheat’) and an elite durum wheat cultivar showing broad phenotypic variation for spike architecture. Besides the previously found gene at the *bh*^*t*^-*A1* locus on the short arm of chromosome 2A, we also mapped two new modifier QTL for spike-branching on the short arm of chromosome 1A, termed *bh*^*t*^-*A2*, and 2BS. Using biparental mapping population and GWAS in 302 diverse accessions, the 2BS locus was highly associated with coding sequence variation found at the homoeo-allele of *TtBH*-*B1* (*bh*^*t*^-*B1*). Thus, RILs that combined both *bh*^*t*^-*A1* and *bh*^*t*^-*B1* alleles showed additive genetic effects leading to increased penetrance and expressivity of the supernumerary spikelet and/or mini-spike formation.

**Supplementary Information:**

The online version of this article (10.1007/s00122-020-03743-5) contains supplementary material, which is available to authorized users.

## Introduction

The spike of *Triticeae* (grass tribe that includes wheat, barley, and rye) is believed to have evolved from a more primitive grass inflorescence known as panicle-like or a compound spike through reductional processes and events (Endress 2010; Kellogg et al. 2013; Vegetti and Anton 1995). As a result, the spike of *Triticeae* is a non-branching inflorescence where spikelets are directly attached to the central axis of the inflorescence (also known as rachis) in a distichous phyllotaxis (Koppolu and Schnurbusch 2019). In addition to the evolutionary processes and events during the formation of grass inflorescences (Poursarebani et al. 2020), the inflorescence morphology of cereal crops has also been altered during the process of crop domestication (Doebley et al. 2006; Piperno 2017; Zhu et al. 2013). Some of the major agronomic traits that were altered during the domestication of wheat are rachis fragility, glume tenacity, and spike threshability resulting in the acquisition of a modern wheat spike known by a non-fragile rachis with a non-tenacious glume for ease of grain threshability (Salamini et al. 2002).

Since its domestication, the importance of wheat and its contribution to global food security is undisputed. Thus, to meet the projected demands of a growing human population, increasing wheat grain yield is urgent (Ray et al. 2013; Sakuma and Schnurbusch 2020). However, increasing wheat yield through conventional wheat breeding is becoming challenging as the harvest index (HI) is believed to approaching the maximum (Austin et al. 1980; Lin and Huybers 2012; Ray et al. 2012; Rose and Kage 2019). Therefore, understanding the evolutionary genetics and developmental basis of the wheat spike is very essential for future yield increase.

Major examples of the advances made in understanding wheat spike development and architecture were the identification of the dominant *Q* locus (Simons et al. 2006) and the branch-suppressing genes: *TtBH/WFZP* and wheat *TEOSINTE BRANCHED 1 (TB1)* (Dixon et al. 2018; Dobrovolskaya et al. 2015; Poursarebani et al. 2015). The *Q*/*q* alleles are involved in spike morphogenesis by suppressing rachilla outgrowth, thereby limiting the floret number within the spikelet. Hence, mutants of the *Q* gene are characterized by extra florets through rachilla extension (Debernardi et al. 2017; Greenwood et al. 2017). Similarly, the loss-of-function of the maize ortholog of the *Q/q* gene, *INDETERMINATE SPIKELET 1* (*IDS1*), led to the conversion of the determinate maize spikelet meristem (SM) to an indeterminate SM, thereby producing an indeterminate number of florets instead of two florets in maize tassel spikelet (Chuck et al. 1998). Recently, the wheat *TB1,* which is also the ortholog of the maize *TEOSINTE BRANCHED1 (TB1),* was found to coordinate the formation of spikelets during the vegetative to floral transition by altering the expression of meristem identity genes on a dosage-dependent manner (Dixon et al. 2018).

On the other hand, *TtBH/WFZP*, which is a wheat ortholog of the maize *BRANCHED SILKLESS 1 (BD1)* or rice *FRIZZY PANICLE (FZP),* is an AP2/ERF transcription factor controlling SM identity in diverse grass species including wheat (Chuck et al. 2002; Derbyshire and Byrne 2013; Dobrovolskaya et al. 2015; Komatsu et al. 2003; Poursarebani et al. 2015). Spike-branching tetraploid wheat mutant is known by the common name ‘Miracle wheat.’ The spike-branching in ‘Miracle wheat’ is characterized by the development of small-sized secondary mini-spikes, similar to branches, directly arising from the rachis node by replacing the spikelets. So far, the gene underlying spike-branching wheat mutants, i.e., *TtBH* and *WFZP*, has been identified in durum and bread wheat, respectively (Dobrovolskaya et al. 2015; Poursarebani et al. 2015). Sequence analysis of ‘Miracle wheat’ accessions also revealed that most of the spike-branching ‘Miracle wheat’ lines carry a mutant form of an ancient *q* allele (*q*^*del*^-*5A)* (Wolde et al. 2019b), suggesting that ‘Miracle wheats’ have mutations both in the SM identity (*TtBH*) and determinacy gene (*Q/q*). Even though the effect of *q*^*del*^-*5A* on spike-branching is not yet clear, results from mutant analyses of the *compositum 2* (*com2*), barley ortholog of *TtBH/WFZP,* showed that the barley *IDS1*, i.e., the *Q*/*q* gene in wheat, is a putative downstream target of *COM2,* which suggests likely interaction of *TtBH/WFZP* and *Q/HvIDS1* during spike development. Therefore, *TtBH/WFZP* and *IDS1/Q* double mutants are essential to further analyze the genetic interaction effect of these two genes during spike morphogenesis.

Even though the major allele underlying ‘Miracle wheat’ has been identified (Poursarebani et al. 2015), previous studies have suggested that spike-branching in wheat is a quantitative trait with the major recessive gene mapped to chromosome 2AS (Echeverry-Solarte et al. 2014; Klindworth et al. 1990; Peng et al. 1998; Sun et al. 2009). Indeed, the gene underlying the QTL on 2AS in tetraploid ‘Miracle wheat’ is *TtBH*-*A1* (Poursarebani et al. 2015). Sequence analysis of *TtBH*-*A1* from a collection of ‘Miracle wheat’ and other tetraploid wheat accessions revealed only a single allele with an amino acid substitution in the AP2/ERF domain of *TtBH*-*A1* (Poursarebani et al. 2015).

In this study, we aimed to map and identify new loci involved in spike-branching using a biparental F7 mapping population and GWAS based on 302 core tetraploid wheat accessions. Our results show that *bh*^*t*^-*B1*, the homoeo-allele of *bh*^*t*^-*A1*, or a very closely linked other gene with similar function modifies spike-branching in tetraploid wheat. Although the *q*^*del*^-*5A* allele from the ‘Miracle wheat’ parent (TRI 19165) segregates in the mapping population, no QTL for spike-branching could be mapped to chromosome 5A harboring the *q*^*del*^-*5A* allele, suggesting that the *Q*/*q*^*del*^-*5A* alleles are not directly involved in spike-branching. However, we found an epistatic interaction between *bh*^*t*^-*A1* and *q*^*del*^-*5A* affecting spike length. Although we did not measure the length of the lateral mini-spike-like branches in the mapping population, it is very likely that the observed epistatic interaction between *bh*^*t*^-*A1* and *q*^*del*^-*5A* is likely to affect the length of the lateral branches. Taken together, our results indicate that both *bh*^*t*^-*A1* and the region around *bh*^*t*^-*B1* play important roles in tetraploid wheat spike architecture. Since the homoeologous *q* gene on 5B does not encode for a full-length protein, ‘Miracle wheats’ have a mutation in the SM identity and determinacy genes, i.e., *bh*^*t*^/*q*^*del*^-*5A*, indicating that they are important to further elucidate the functional relationship between TtBH and Q/q^del^-5A protein during spike development to better understand the genetics and molecular basis of wheat spike morphogenesis.

## Materials and methods

### Development of the F7 Recombinant inbred lines (RILs)

One hundred forty-six F7-derived RILs were developed through single-seed descent (SSD) from an F2 population derived from a cross between Bellaroi (an elite durum wheat variety with spring growth habit) and TRI 19165 (a ‘Miracle wheat’ and winter type). Since the mapping population segregated for winter/spring growth habit, all RILs with winter growth habit were excluded from the mapping population by growing all the F2 plants without vernalization in the greenhouse. Those that were able to complete their life cycle and give grains without vernalization were all spring types (*n* = 146) and were used for this study. The RILs were evaluated under field conditions for two consecutive years in 2014 and 2015 growing seasons in three different environments (IPK14, IPK15, and HAL15) in Germany. In 2014, the F7-derived RILs were evaluated in Gatersleben (IPK14), 51.49° N and 11.16° E, Germany. In the following season, i.e., 2015, the F8-derived RILs were evaluated in two environments: Gatersleben (IPK15) and Halle/Saale (HAL15), Germany. All field evaluations were conducted on 3.75 m^2^ plots in a randomized complete block design (RCBD) in two replications. Three hundred grains per m^2^ were sown in rows spaced at 20 cm. Plants in all locations were fertilized according to the soil conditions in each environment. Similarly, herbicides and fungicides were also applied to control weeds and fungal infestations. Besides the three environments, F8-RILs were also evaluated under greenhouse conditions at IPK (GH15) without a replication.

### Plant materials for the Genome-Wide Association Study (GWAS)

Three hundred two tetraploid wheat accessions from a core collection comprising one hundred seventy-seven free-threshing durum wheat, thirty-one hulled emmer wheat, twenty-four rivets or cone wheat, twenty-seven ‘Miracle wheat,’ eleven wild emmer wheat, and thirty-two other tetraploid wheat were obtained from the Leibniz Institute of Plant Genetics and Crop Plant Research (IPK). All accessions were grown under controlled long-day conditions in the greenhouse at IPK. While the spring types were vernalized for 4 weeks at 4 °C, the winter and intermediate types were vernalized for 8 weeks. All plants received equal amounts of fertilization and watered uniformly throughout the growth period.

### Phenotyping

Additional spikelet number per spike due to supernumerary spikelets (SS) and/or genuine spike-branching from 10 to 15 sampled spikes from the middle row of each plot from each environment was used for QTL mapping in the RILs. At maturity, spike length was also measured in the field from at least five random plants from the middle rows of each plot. Besides the additional spikelet number per spike, the total number of spikelets (the sum of additional spikelet number per spike and the remaining primary spikelet) was also used for the epistatic interaction analysis. Phenotyping of accessions for GWAS analysis was performed based on a scale from 0 to 4, where 0 was given to a non-branching or wild-type spike and 4 to the strongly spike-branching phenotype.

### DNA extraction and genotyping

Genomic DNA from all RILs at F7 generation was isolated based on the modified CTAB method as described by Doyle (1990). The final concentration was measured, and samples were used for CAPS marker analysis as well as for Genotyping-By-Sequencing (GBS) library preparation following a novel two-enzyme genotyping-by-sequencing approach (Poland et al. 2012).

### Genotyping of the mapping populations

DNA markers from RILs were generated by genotyping-by-sequencing (GBS) following the two-enzyme approach (Poland et al. 2012). Similarly, GBS was used to genotype the GWAS population. Adapters were trimmed from reads with cutadapt version 1.8.dev0 (Martin 2011). Trimmed reads were mapped to the chromosome-shotgun assemblies of bread wheat cultivar Chinese Spring (The International Wheat Genome Sequencing Consortium (IWGSC) 2014) with BWA mem version 0.7.12 (Li 2013), converted to BAM format with SAMtools (Li et al. 2009) and sorted with Novosort (Novocraft Technologies Sdn Bhd, Malaysia, http://www.novocraft.com/). Multi-sample variant calling was performed with SAMtools version 0.1.19 (Li 2011). The command ‘mpileup’ was used with the parameters ‘C50 –DV.’ The resultant VCF file was filtered with an AWK script provided as Text S3 by Mascher et al. (2013). Only biallelic SNPs were used. Homozygous genotype calls were set to missing if their coverage was below 1 or their genotype quality was below 3. Heterozygous genotype calls were set to missing if their coverage below 4 or their genotype quality was below 10. SNP was discarded (1) if its quality score was below 40, (2) its heterozygosity was above 20%, (3) its minor allele frequency was below 10%, or (4) had more than 66% missing data. Genotype calls were filtered and converted into genotype matrix with an AWK script available as Text S3 of Mascher et al. (2013). Chromosomal locations and genetic positions were taken from the population sequence (POPSEQ) data (Chapman et al. 2015).

### Linkage map construction

The genetic linkage map of RILs was constructed using Joinmap 4.0 (Stam 1993). After removing all the distorted markers based on goodness-of-fit using a Chi-squared test, 427 SNP markers were used for the linkage map construction. Regression and maximum likelihood mapping algorithms were followed to produce the map. Linkage groups were determined using Haldane’s mapping function. The maximum distance of 50 cM was used to determine the linkage between two markers. The maps were drawn using Map chart version 2.3 (Voorrips 2002).

### Development of CAPS marker

The diagnostic CAPS marker linked with the *bh*^*t*^-*A1* allele has been previously described in the supporting information (Poursarebani et al. 2015). Similarly, a CAPS marker was developed based on SNP located -54 bp upstream to the start of the coding sequence of the homoeologous *TtBH*-*B1* gene. Enzyme *XhoI* from New England Biolabs®, USA, was used for restriction digestion analysis. Primers WFZP_2B_F2 (Dobrovolskaya et al. 2015) and Tafzp_2B_R1 were used to amplify the 5′UTR including the coding region (Supplementary File 1). Touchdown PCR was used for amplification. The conditions were as follows: 95 °C/3 min, 8 cycles of 95 °C/30 s, 67 → 60 °C/30 s (− 1 °C in each step), then 36 cycles of 95 °C/30 s, 62 °C/30 s, 72 °C/1 min and 72 °C/10 min. Restriction digestion with *XhoI* was as follows: 4 μl of the PCR product; 5 units of *XhoI* (0.5 μl); 0.15 μl BSA (Bovine Serum Albumin), 2 μl of reaction buffer, and 3.35 μl of water were mixed and incubated overnight at 37^0^c. The digested product was analyzed on a 2% agarose gel.

### QTL mapping

Phenotypic data from all 146 RILs from the three different environments (IPK14, IPK15, and HAL15) as well as from the greenhouse (GH15) were used for QTL mapping using Genstat 17. First, single environment–single trait linkage analysis was performed to map QTL associated with SS in each environment. A step size of 10 cM, minimum cofactor proximity of 50 cM, a minimum separation of selected QTL of 30 cM, and a genome-wide significance level (alpha) of 0.05 were used for QTL analysis. Based on the mixed-model approach, the whole genome was scanned first using a simple interval mapping (SIM) approach. Then, based on the SIM result, cofactors were selected for composite interval mapping (CIM). The final QTL model was selected using the backward selection on the selected cofactors, where QTL boundaries (lower and upper), QTL effect and phenotypic variance explained (PVE) by QTL were determined. Under the option of a single trait and multiple environments analysis toolbox, a similar setup was followed to check whether there was QTL by environment interaction. The epistatic interaction was mapped using QTL network version 2 (Yang et al. 2008). The GWAS was performed following the compressed mixed linear model (Zhang et al. 2010) implemented in the GAPIT R package (Lipka et al. 2012). About 25,166 SNP markers were used for the analysis.

### Expression analysis

Total RNA was extracted from four different RILs at three spike developmental stages, i.e., glume primordium (GP), floret primordium (FP), and terminal spikelet (TS) stages. We selected RIL 769-3-21, RIL 7769-5-6, RIL 7769-3-22, and RIL 7769-4-38 for the analysis (Table 1).

**Table 1:** Recombinant Inbred Lines (RILs) selected for expression analysis

RIL	Genotype	Phenotype
RIL 7769-3-21	*bh*^*t*^-*A1*/*bh*^*t*^-*B1 (*aabb)	Strongly branching
RIL 7769-5-6	*BH*^*t*^-*A1*/*BH*^*t*^-*B1* (AABB)	Wild type (non-branching)
RIL 7769-3-22	*BH*^*t*^-*A1*/*bh*^*t*^-*B1 (*AAbb)	Wilde type (non-branching)
RIL 7769-4-38	*bh*^*t*^-*A1*/*BH*^*t*^-*B1* (aaBB)	Supernumerary spikelet (SS)

RIL 7769-3-21 (genotype: aabb) was selected because both homoeo-alleles, i.e., *bh*^*t*^-*A1 (aa)* and *bh*^*t*^-*B1(bb)* were inherited from the mutant parent, TRI 19165. The spike was strongly branching in all environments. RIL 7769-5-6 (genotype: AABB), was selected because both homoeo-alleles were inherited from the wild-type parent, Bellaroi. As a result, the spike was wild type. RIL7769-3-22 (genotype: AAbb) was selected because the *BH*^*t*^-*A1* (AA) allele was inherited from Bellaroi, while the *bh*^*t*^-*B1* (bb) allele was from TRI 19165. Since it carried the functional *BH*^*t*^-*A1* allele from Bellaroi, it did not show any spike-branching. Moreover, RIL 7769-4-38 (genotype: aaBB) was selected because the *bh*^*t*^-*A1* (aa) allele was inherited from TRI19165, while the *BH*^*t*^-*B1* (BB) allele was inherited from Bellaroi. Spikes of this allelic combination showed only a few SS. Plants were grown under controlled long-day conditions: 16/8 h day/night; and 19/17 °C day/night temperatures for 15 days. After 15 days of germination and seedling establishment, plants were vernalized for about 4 weeks at 4 °C. After 1 week of hardening at 15/12 °C day/night temperature, all seedlings were transferred to 20/17 °C day/night temperature and allowed normal development. Spike development stages, based on Kirby cereal development guide handbook (Kirby and Appleyard 1984), were frequently checked at after 4 leaf stages and beyond. The whole inflorescence was removed at the right stages by carefully dissecting the plant under a light microscope, and samples were frozen in liquid nitrogen immediately until RNA isolation. Total RNA was isolated using TRIzol® RNA isolation reagents (Thermo fisher scientific Catalog # 15596018). After quantification and quality check, the total concentration was adjusted to 50 ng/μl for all samples and the first-strand cDNA was synthesized with oligo (dT) primer and Superscript™ III (Invitrogen, Life Technologies) according to the manufacturer’s protocol.

Gene-specific primers qBh_2A_F2 and qBh_2A_R2 for *TtBH*-*A1* and qBh_2B_F3 and qBh_2B_R3 for *TtBH*-*B* were designed from the 3′UTR region of each gene. Wheat *Actin* gene primers were taken from Distelfeld et al. (2009), which was used as an internal reference. All primers for the expression analysis were diluted to a final concentration of 0.5 pM. Before proceeding to the expression analysis, the first-strand cDNA was diluted 100 times in RNA-free DEPC-treated water. For each reaction, 2 µl of the forward primer, 2 μl of reverse primer, 1 µl cDNA, and 5 µl SYBR^®^ Green PCR Master Mix (Applied Biosystems, Warrington, UK) were used. For each gene, four technical and two to three biological replications were used. The ABI 7900HT Fast Real-Time PCR system (Applied Biosystems, Foster City, USA) was used for the analysis. The cycle threshold (*C*_t_) values were extracted using SDS2.4 software from the ABI 7900HT Fast Real-Time PCR system. PCR efficiency (*E*) was calculated using LinReg PCR (Version 7.5). The expression value was normalized using *Actin* as an internal reference gene.

### Sequencing and sequence analysis

We sequenced homoeologous genes from 116 *T. turgidum* and *T. durum* (*TtBH*-*A1, TtBH*-*B1*). The putative promoter regions of *TtBH*-*A1* (1.3 kb) and *TtBH*-*B1* (2.1 kb) from 39 canonical and 28 spike-branching accessions were also sequenced. Most of the *T. turgidum* and *T. durum* were selected from the core collection that was used for GWAS (Supplementary File 1). All the genome-specific primers used for the amplification and sequencing are listed in Supplementary File 1. DNA sequence analysis and multiple alignments were conducted using Geneious software version 6 (Kearse et al. 2012).

## Results

### Phenotypic expression of spike-branching in the mapping population

**Fig. 1:**
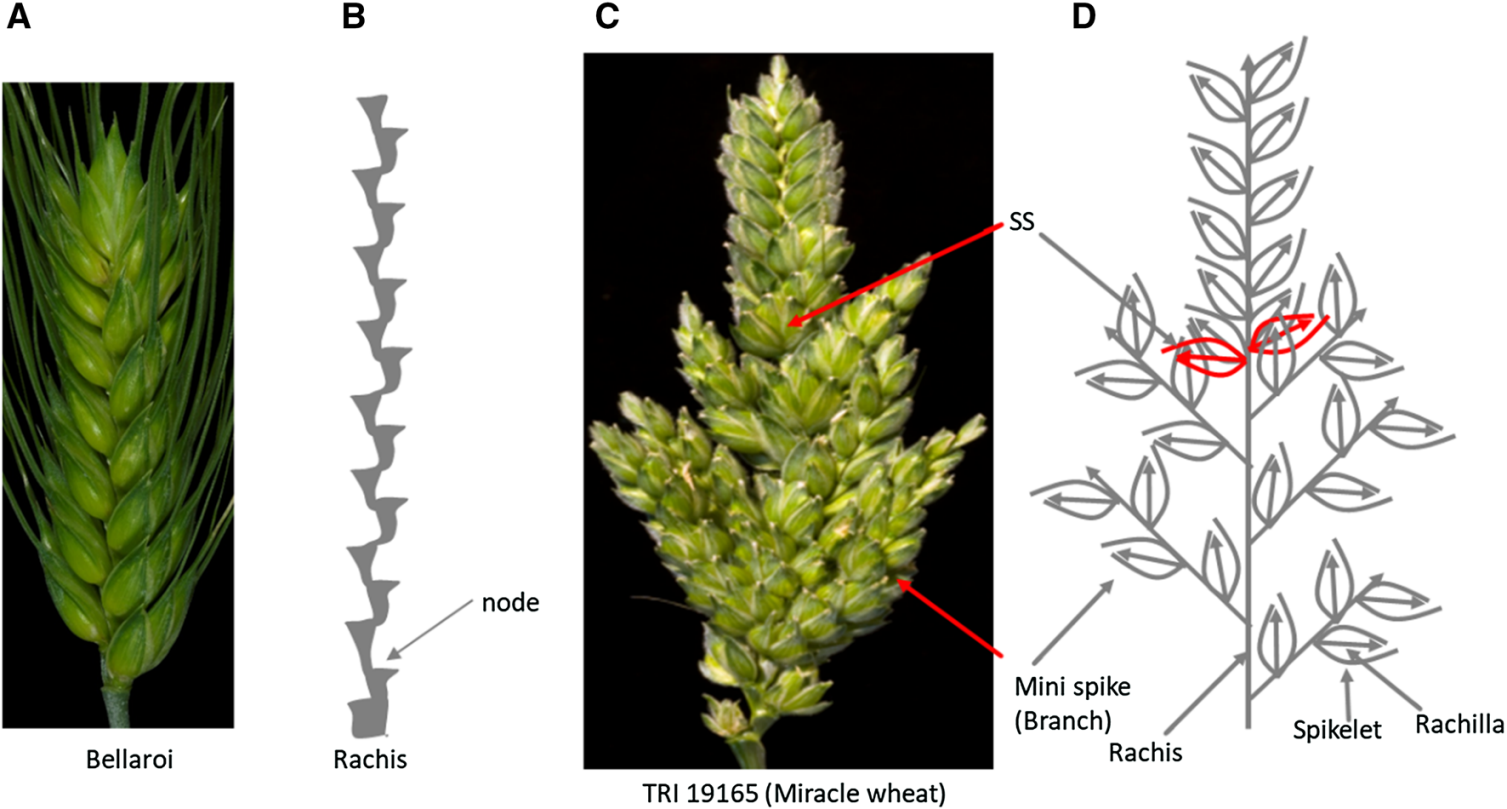
Spike morphological differences between parent Bellaroi (**a**) and TRI 19165 (**c**). For clarity, awns were removed from TRI 19165. Spike from Bellaroi shows a canonical or unbranched spike, where each node or spikelet is arranged in a distichous order along the central axis, the rachis (**b**). The mini-spike-like structures (branches) arising from the nodes of the bottom half the rachis of TRI 19165 (**c**) and schematic sketch depicting spike-branching in ‘Miracle wheat’ (**d**). Red colored spikelets show supernumerary spikelets (SS) sharing the same rachis node (color figure online)

Spike morphologies of the parents of the mapping population, i.e., cv. Bellaroi and TRI 19165 (‘Miracle wheat’), are shown in Fig. 1a, c, respectively. The sessile spikelets in a standard spike of Bellaroi are attached directly onto the rachis node in a distichous arrangement (Fig. 1a, b). However, most of the bottom nodes in ‘Miracle wheat’ spikes do not carry sessile spikelets but instead form ‘mini-spikes’ like branches carrying sessile spikelets by their own (Fig. 1c, d). Previously, we have identified and characterized the ‘Miracle wheat’ allele (*bh*^*t*^-*A1)* from spike-branching tetraploid wheat (Poursarebani et al. 2015; Wolde et al. 2019a). Because spike-branching appeared to be a quantitative trait, we conducted the current study to identify other loci controlling spike-branching in tetraploid wheat.

**Fig. 2:**
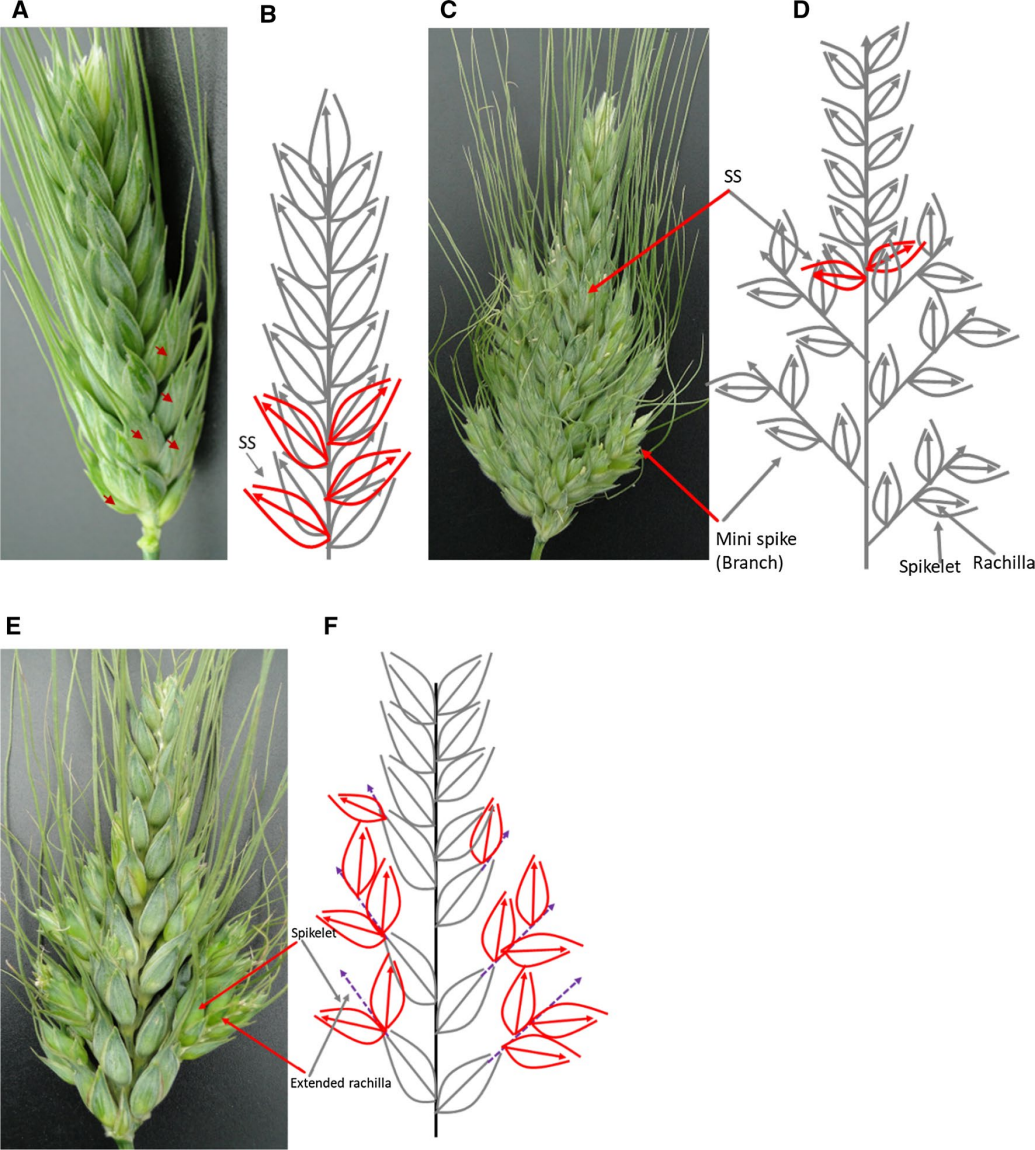
Summary of the phenotypic variation of spike-branching in the mapping population. **a** Additional or supernumerary spikelets (SS, arrowheads) sharing the same rachis node with the primary spikelet. **b** Schematic sketch depicting SS (red) sharing the same rachis node with the primary spikelet (Black). Genuine spike-branching appearing from rachis node in place of spikelets (**c**, **d**) and extension of the rachilla (broken arrowhead) or the conversion of rachilla into rachis carrying spikelets in place of florets (**e**, **f**, red spikelets). No rachis-branching was observed in this line (RIL #7769-4-78), i.e., the only RIL where the primary spikelets appeared in a distichous order on the central axis without mini-branch or SS formation; except the formation of new spikelets on the extended rachilla in place of the florets. This type of phenotype appeared exceptionally in one RIL, i.e., RIL #7769-4-78 (color figure online)

As expected, the RILs used in this study generally showed three different types of spike phenotypes (Fig. 2). The first one was the appearance of SS (Fig. 2a, b). The second type was the appearance of ‘mini-spikes’ from the bottom half of the spike resembling the ‘Miracle wheat’ phenotype (Fig. 2c, d). The third type was the extension of the rachilla, and/or partial conversion of the rachilla to rachis leading to the appearance of alternated spikelets and florets (Fig. 2e, f). However, out of the 146-mapping population only one RIL, i.e., 7769-4-78, showed the rachilla extension phenotype. Although the three phenotypic classes were detected, all led to the appearance of additional spikelets per spike. Therefore, the number of supernumerary spikelets (SS) per spike, i.e., additional spikelets per spike (addSPS), was used for mapping spike-branching. The phenotypic data are summarized in Supplementary Tables 1 and 2.

### Genome-wide identification of loci controlling spike-branching in tetraploid wheat

**Fig. 3:**
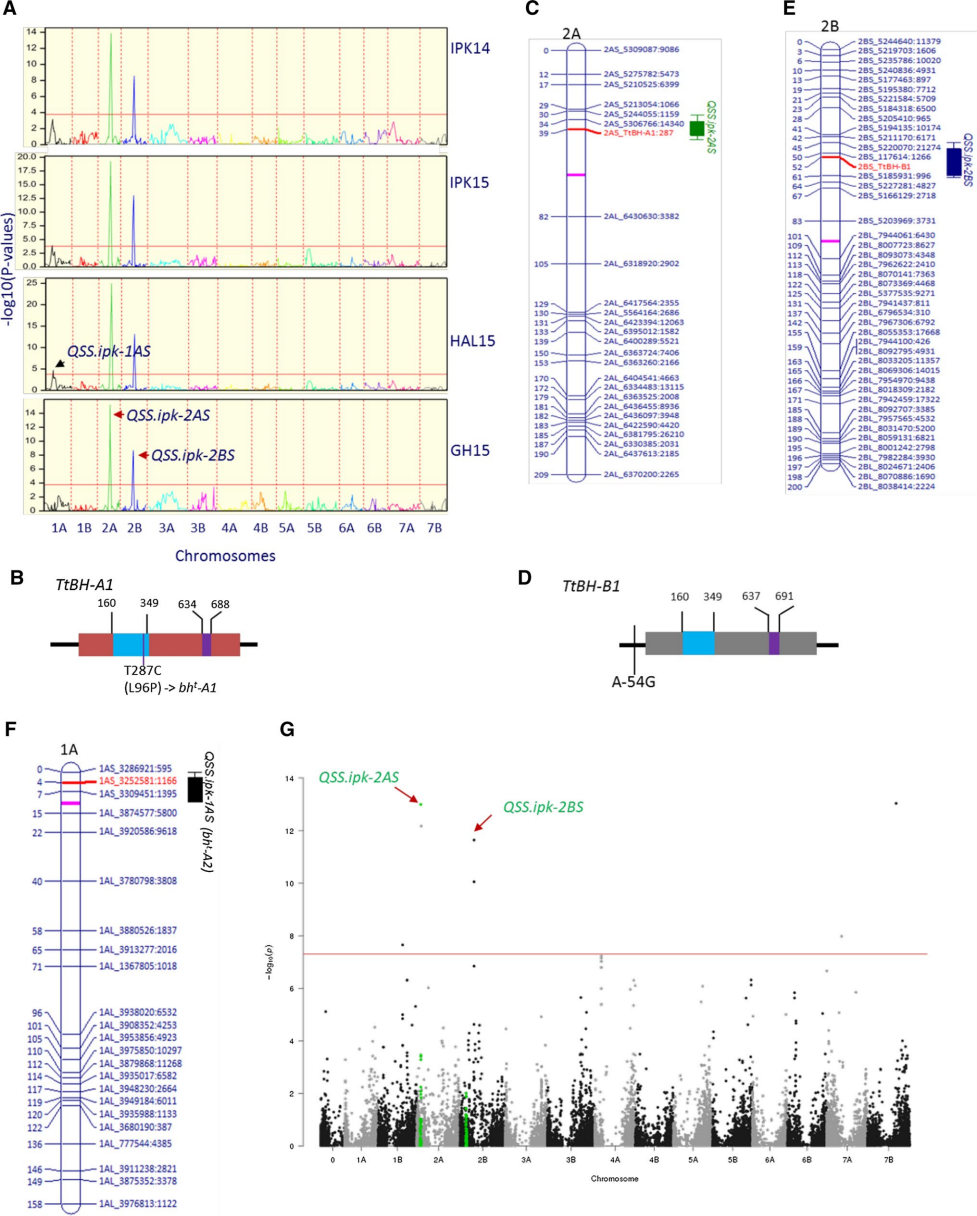
Chromosomal locations of identified QTL. **a** Independent detection of QTL in four different environments: IPK14, IPK15, HAL15, and GH15. *QSS.ipk*-*2AS* and *QSS.ipk*-*2BS* consistently appeared in all environments. **b** *TtBH*-*A1* gene model showing an AP2/ERF DNA-binding domain (blue), and the mutation (T287C) responsible for spike-branching in tetraploid wheat as reported by Poursarebani et al. (2015). The purple bar in the C-terminal region shows a highly conserved region. The *bh*^*t*^-*A1* CAPS diagnostic marker for spike-branching on chromosome 2AS was developed based on the T287C substitution. **c** Chromosomal locations bearing *QSS.ipk*-*2AS.* As shown, *QSS.ipk*-*2AS* was linked with the CAPS marker (red font), indicating that the underlying gene for *QSS.ipk*-*2AS* is indeed *bh*^*t*^-*A1*. **d** Gene model for the homoeo-gene *TtBH*-*B1* showing the conserved AP2/ERF DNA-binding domain (blue) and the conserved C-terminal region (purple). The A-54G SNP was used for the development of the CAPS marker to differentiate the parental alleles, i.e., Bellaroi versus TRI 19165. This SNP is located within the putative 5′-UTR region, and all spike-branching tetraploid wheat accessions (*n *= 49) carry the G. From 48 accessions with canonical spike shape, 14 of them also carry the G, while the remaining 34 carry A (Supplementary File 1). **e** Chromosomal locations bearing *QSS.ipk*-*2BS.* As shown, *QSS.ipk*-*2BS* was linked with the *TtBH*-*B1*-derived CAPS marker (red font), indicating that the underlying gene for *QSS.ipk*-*2BS* is most likely allelic or close to *TtBH*-*B1*. **f** The second minor effect QTL was identified on chr. 1AS as *QSS.ipk*-*1AS* or *bh*^*t*^-*A2*. This QTL did not appear in all environments (**a**). **g** GWAS results showing QTL from chr. 2AS and 2BS within 10 Mb around *QSS.ipk*-*2AS* and *QSS.ipk*-*2BS.* Green dots represent gene-derived CAPS markers. Purple lines in **c**, **e** and **f** demarcate the short and long arms of the chromosomes (centromeric region) (color figure online)

To identify loci controlling spike-branching, we mapped SS formation per spike (Fig. 2a, c, e). Following composite interval mapping (CIM) analysis, three QTL, i.e., *QSS.ipk*-*1AS, QSS.ipk*-*2AS,* and *QSS.ipk*-*2BS*, were identified as controlling spike-branching in this mapping population (Fig. 3a). The QTL on chromosome group 2 consistently appeared in all four environments, i.e., IPK14, IPK15, HAL15, and GH15. Since the ‘Miracle wheat’ allele (*bh*^*t*^-*A1*) had already been identified from chromosome 2A short arm (Poursarebani et al. 2015), we genotyped the RILs using diagnostic CAPS markers derived from the *bh*^*t*^-*A1* allele, as well as from the homoeo-allele from chromosome 2B, i.e., *TtBH*-*B1.* Then, we incorporated the two new CAPS markers to the GBS markers and re-run the mapping analysis. Our results showed that the two QTL identified earlier, i.e., *QSS.ipk*-*2AS* and *QSS.ipk*-*2BS,* were closely linked to the corresponding CAPS markers (Fig. 3c, e). Based on our previous results (Poursarebani et al. 2015) and the complete linkage of *QSS.ipk*-*2AS* with the *bh*^*t*^-*A1* CAPS marker (Fig. 3c), we concluded that *QSS.ipk*-*2AS* was indeed the *bh*^*t*^-*A1* allele. Tight linkage of the *TtBH*-*B1*-derived CAPS marker with *QSS.ipk*-*2BS* also suggests that *TtBH*-*B1* or a very closely linked gene was responsible for the phenotypic variance at *QSS.ipk*-*2BS,* further implying that *QSS.ipk*-*2AS* and *QSS.ipk*-*2BS* are likely to be the homoeoloci, *bh*^*t*^-*A1* and *bh*^*t*^-*B1*, respectively. The QTL on chromosome 1AS, *QSS.ipk*-*1AS,* had a significant effect on the spike-branching phenotype in one environment (HAL15); but remained under the significance level in the other three environments. We, therefore, decided to term this new locus as *bh*^*t*^-*A2*. The phenotypic variance explained by these three QTL is shown in Supplementary Table 3.

Furthermore, we performed a GWAS analysis based on 302 tetraploid wheat including 27 ‘Miracle wheat’ accessions. First, we looked at the diversity of different wheat species within the dataset which enabled us to carefully analyze population structure. The principal component analysis (PCA) revealed that the spike-branching wheats are distinctly grouped from all other accessions (Supplementary Figure 1). Despite the small number of spike-branching accessions in the data set, the distinct position of spike-branching accessions within the data set allowed us to conduct GWAS using the linear model with kinship information.

The GWAS analysis revealed two QTL on chromosome 2A and 2B (Fig. 3g). However, the slight deviation of the lead marker (black dot) from the *bh*^*t*^-*B1* derived CAPS marker (green) could partly be due to the linkage disequilibrium (LD) of the lead marker as a result of different factors including population structure and genetic linkage. Taken together, the biparental QTL mapping and GWAS analysis suggest that the allelic variation close to *bh*^*t*^-*B1* is highly linked with *QSS.ipk*-*2BS*.

**Fig. 4:**
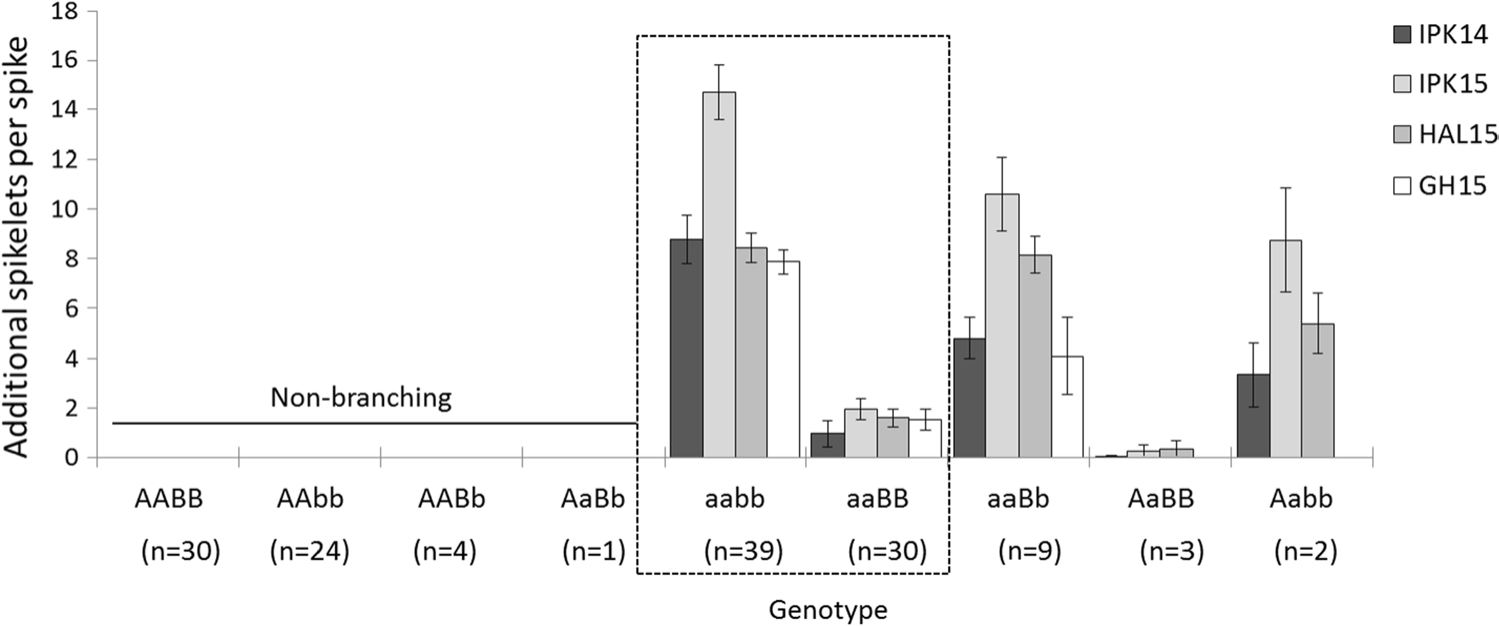
Phenotypic comparison after grouping the RILs based on CAPS markers derived from the homoeo-alleles (see Fig. 2b, d). All small letters indicate alleles from ‘Miracle wheat’ accession TRI 19165. The first four groups, i.e., AABB, AAbb, AABb, and AaBb, carry the functional *TtBH*-*A1* allele from Bellaroi and did not show any type of spike-branching in any of the environments. The remaining five groups showed differential spike-branching with a varying degree of expressivity. The aabb group (*n *= 39) carrying both alleles (*bh*^*t*^-*A1, bh*^*t*^-*B1)* from TRI19165 showed spike-branching with a higher degree of expressivity in all environments. Interestingly, the aaBB group (*n* = 30), carrying only the *bh*^*t*^-*A1* allele from TRI19165 and the B allele (*BH*^*t*^-*B1*) from Bellaroi showed reduced expression of spike-branching indicating its role as a branch suppressor. IPK14, IPK15, HAL15, and GH15 are environments

To further test the effect of the different homoeo-allelic combinations on spike-branching, we divided the F7-RILs into nine genotypic groups using the CAPS markers derived from *bh*^*t*^-*A1* and *bh*^*t*^-*B1.* The genotypes were designated as AABB (those RILs carrying both homoeo-alleles from Bellaroi), AABb (those RILs carrying the A copy from Bellaroi, but heterozygous for the B copy), AaBB (those RILs heterozygous for the A copy, but carrying the B copy from Bellaroi), AaBb (those RILs heterozygous for both copies), Aabb (those RILs heterozygous for the A copy, but carrying the B copy from TRI 19165), aaBB (those RILs carrying the A copy from TRI 19165, and the B copy from Bellaroi), aaBb (those RILs carrying the A copy from TRI 19165, but heterozygous for the B copy), AAbb (those RILs carrying the A copy from Bellaroi and the B copy from TRI 19165), and aabb (those RILs carrying both homoeo-alleles from TRI 19165). The summary of the phenotypic comparison among the nine groups across the four different environments is shown in Fig. 4. Due to the functional allele from Bellaroi for the 2AS QTL (AA), the first four groups of RILs, i.e., AABB, AAbb, AABb, and AaBb, did not show any form of the spike-branching phenotype across all environments. This suggests that the wild type allele from Bellaroi was sufficient for maintaining the canonical spike form. On the other hand, RILs carrying both homoeo-alleles from ‘Miracle wheat,’ i.e., *aabb,* showed more SS and/or genuine spike-branching as compared to those RILs carrying only the recessive A allele (aaBB) that only showed SS formation (Fig. 4, boxed). This also suggests a branch modifying role of the BB genome allele from TRI 19165, i.e., *bh*^*t*^-*B1*. Because the suppressive effect of the wild-type (i.e., the BB allele from Bellaroi) did not completely abolish SS formation (Fig. 4), the wild-type allele from the AA genome plays a major role in maintaining the canonical spike form in tetraploid wheat (Poursarebani et al. 2015). Genome-wide epistatic interaction mapping also revealed a significant additive-by-additive epistatic effect between *bh*^*t*^-*A1* and *bh*^*t*^-*B1* (Supplementary Table 4).

**Fig. 5:**
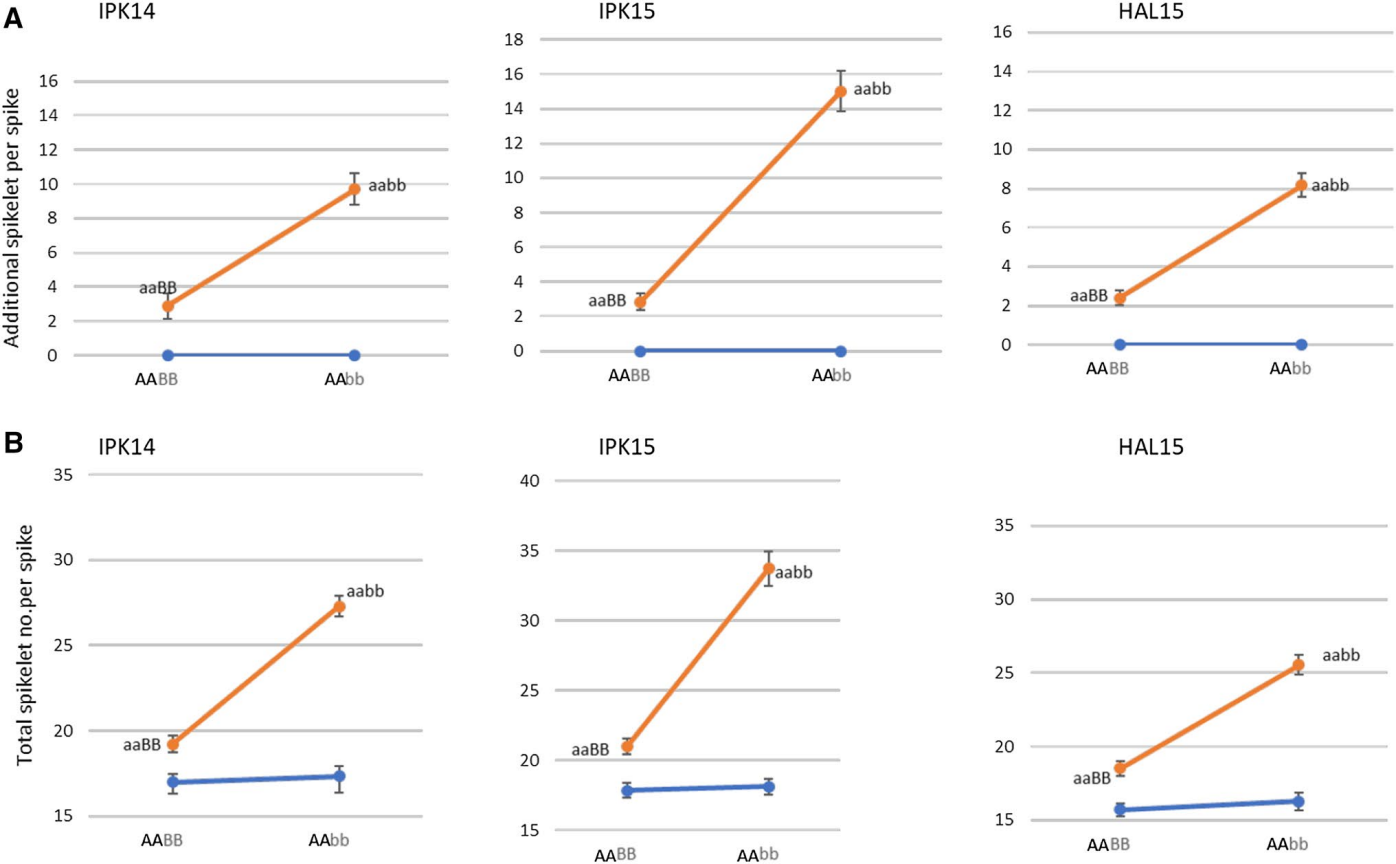
Reaction norms depicting epistatic interactions between ‘Miracle wheat ‘homeo-alleles (aa and bb) affecting additional spikelets per spike (**a**) and total spikelets per spike (**b**). Epistasis is deduced from the differences of the slopes of the lines connecting each group (orange vs blue). Genotypic group trait values for RILs with AABB, AAbb, aaBB, and aabb are the mean trait values computed from three different environments (IPK14, IPK15, and HAL15) (color figure online)

Next, we plotted the two-dimensional graphs of the phenotypic response (the reaction norm) of RILs with genotype AABB, AAbb, aaBB, and aabb across three different environments (Fig. 5). Besides the suppression effect of an allele from Bellaroi (BB) on the SS and/or spike branch formation, clearer epistatic interaction was also deduced from the differences observed in the slopes of the lines connecting each group (orange vs blue). Taken together, these results indicate that phenotypic variation for spike-branching in the RILs was mainly controlled by the genetic loci containing the homoeo-alleles *bh*^*t*^-*A1* and *bh*^*t*^-*B1*.

### Effects of *QSS.ipk*-*2A, QSS.ipk*-*2B*, and *Rht*-*B1b* on spikelet fertility, grain number, and grain weight

**Fig. 6:**
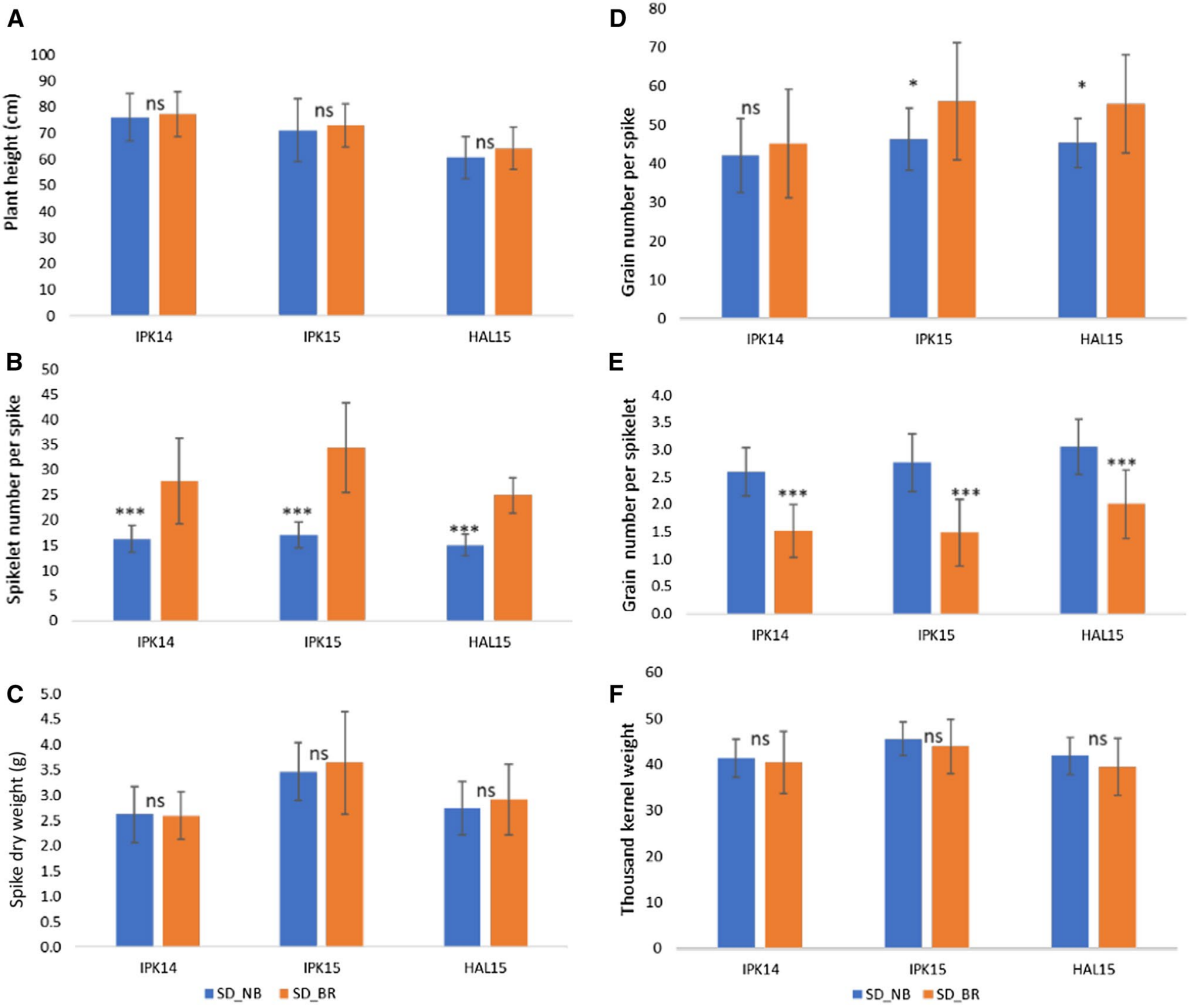
Comparison of selected semi-dwarf RILs with and without QSS.ipk-2A and QSS.ipk-2B (*n * = 13, and 16, respectively) based on three different environments: IPK14, IPK15, and HAL15. (**A**) Plant height (cm) of selected semi-dwarf RILs. (**B**) Spikelet number per spike. (**C**) Spike dry weight (g) per spike. (**D**) Grain number per spike. (**E**) Grain number per spikelet. (**F**) Thousand kernel weight (g). SD_NB, Semi-Dwarf, Non-Branching RILs; SD_BR, Semi-Dwarf, Branching RILs. Error bars are mean ± SD. Significance level between group means was calculated based on unpaired two-tailed Student’s t test at p = 0.05, * and *p* = 0.001, ***. ns, nonsignificant

Compared to tall wheat varieties, semi-dwarfing of the modern wheat varieties with *Reduced height* (*Rht*) genes partition more dry matter to the developing spike, resulting in increased grain number per spike (Brooking and Kirby 1981; Flintham et al. 1997; Miralles et al. 1998; Youssefian et al. 1992). Bellaroi is a semi-dwarf modern durum wheat variety with a reduced height gene, i.e., *Rht*-*B1b* allele. The semi-dwarf RILs were selected based on the plant height QTL that was mapped to a region harboring *Rht*-*B1b* (Supplementary Figure 2). Thus, we compared 13 semi-dwarf RILs carrying *QSS.ipk*-*2A* (*bh*^*t*^-*A1*) and *QSS.ipk*-*2B* (*bh*^*t*^-*B1*) to those without positive alleles at *QSS.ipk*-*2A* and *QSS.ipk*-*2B* (*n* = 16) to better analyze the effect of the *Rht*-*B1b* allele on spikelet fertility and grain number as the spikelet number increases. Despite increased spikelet number due to SS formation and/or mini branches, which resulted in a significantly higher number of grains per spike (Fig. 6b), spikelet fertility or grain number per spikelet was decreased (Fig. 6e). This suggests that increasing sink or spike size alone is not sufficient without solving the problem of spikelet infertility, which is also termed as floret abortion (Sakuma and Schnurbusch 2020). Importantly, we did not find significant differences in kernel weight between the two groups in all three locations (Fig. 6f), which suggests the possibility of increasing wheat yields by increasing spikelet number without significantly affecting the required kernel weight in a semi-dwarfed background.

### Both homoeo-genes were expressed during early stages of spike development

**Fig. 7:**
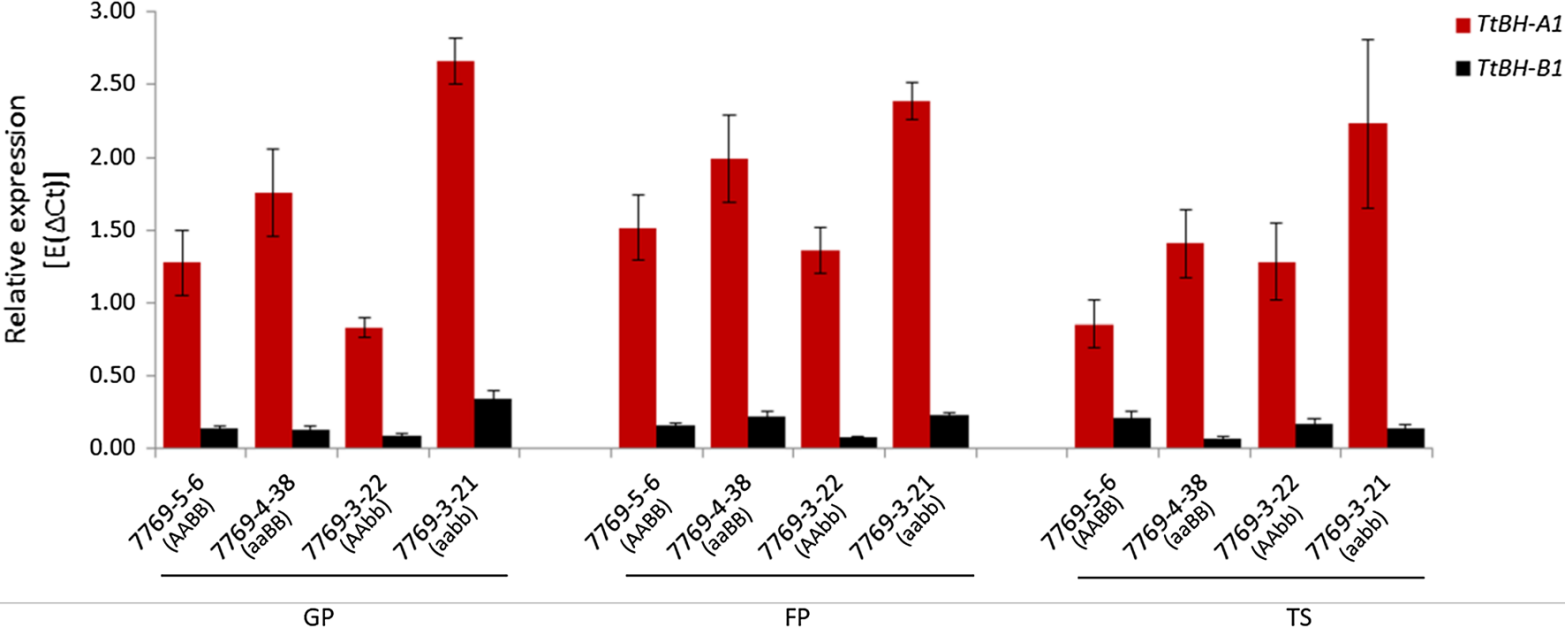
mRNA levels of *TtBH*-*A1* and *TtBH*-*B1* in selected spike stages. The analysis was performed based on four selected RILs (7769-5-6 (genotype: AABB), 7769-4-38 (genotype: aaBB), 7769-3-22 (genotype: AAbb), and 7769-3-21 (genotype: aabb)) at three spike developmental stages: glume primordia (GP), floret primordia (FP), and terminal spikelet stage (TS). RILs carrying the mutant (aa) allele combination did not show lower transcript levels in comparison with AA, again reconfirming the fact that transcriptional differences among alleles are not causative for the spike phenotype. Instead, the SNP located in the coding region, T287C, conferring the L96P substitution may cause reduced protein functionality. Relative expression of *TtBH*-*A1* and *TtBH*-*B1* was calculated based on ∆*C*_t_ (cycle threshold) values after values were normalized against the Ct values of the reference gene *Actin.* Error bars, ± St. Err. Means were calculated from three biological replicates

To complement mapping results, transcript levels of *TtBH*-*A1* and *TtBH*-*B1* were measured at three spike developmental stages using four RILs selected from the mapping population based on different allelic combinations of the homoeo-alleles (See Materials and Methods). Consistent with the observed QTL effects (Fig. 3a) and group phenotypic effects of the RILs (Fig. 4), our expression analysis also indicated that *TtBH*-*A1* was the higher expressed homoeo-allele, followed by *TtBH*-*B1* (Fig. 7). Taken together, these results further demonstrated that *TtBH*-*A1* is the gene majorly controlling SM identity and maintenance of the canonical spike form in tetraploid wheat.

### Sequence analysis of homoeologous *TtBH*-*A1* and *TtBH*-*B1* genes

To investigate the allelic variation between the two homoeo-genes, we sequenced the homoeologous genes from 116 tetraploid wheat species (*T. turgidum and T. durum*; *TtBH*-*A1*, *TtBH*-*B1*). The AA copy, i.e., *TtBH*-*A1,* of the gene from these species encodes for 299 amino acids, while the BB copy, *TtBH*-*B1,* encodes for 307 amino acids. Both homoeo-genes have a highly conserved AP2/ERF DNA-binding region as well as a conserved motif in the C-terminus region (Fig. 3b, d). Mutations in the conserved regions led to SS formation and/or spike-branching (Dobrovolskaya et al. 2015; Poursarebani et al. 2015). Similarly, the *bh*^*t*^-*A1* or the ‘Miracle wheat’ allele (Fig. 3b and Table 2, tHAP_6) arose due to a non-synonymous substitution (T287C; L96P) in the AP2/ERF coding region of *TtBH*-*A1* (Poursarebani et al. 2015). From the sequenced 116 tetraploid wheat species (*T. turgidum and T. durum*), we did not find any other causative mutation in *TtBH*-*A1* other than the T287C substitution (Table 2). In *TtBH*-*B1* (taking Chinese Spring as the reference genome), three non-synonymous substitutions A491G, A619T, and T785C were identified. Interestingly, all of the spike-branching accessions carry the T785C substitution, suggesting that T785C is most likely linked with an increased phenotypic expression of SS formation and/or spike-branching in RILs, which have combined both alleles, i.e., aabb, from TRI19165 (Fig. 4). Of course, the causative phenotypic effect of the mutation from *TtBH*-*B1* cannot be easily deduced because of the masking effect, especially when the *TtBH*-*A1* is functional. This was exactly the case in the tHAP_5 haplotype, where the spike was normal and did not show any SS, even though the TtBH-B1 protein is non-functional as a result of early stop codon due to frameshift mutation incurred as a result of six base pair deletion (Table 2). This result further confirms that *TtBH*-*A1* is sufficient to maintain the canonical spike architecture regardless of the allelic status at *TtBH*-*B1*. Hence, all accessions carrying the tHAP_1, 2, 3, and 5 showed a non-branching spike due to a functional *TtBH*-*A1* allele (Table 2, Fig. 4). Bellaroi, which was used as a non-branching parent for the mapping population, was grouped in tHAP_3, while TRI 19165 was grouped in tHAP_6.

**Table 2:** Haplotype analysis based on the coding sequence of homoeologous genes in tetraploid wheats

Haplotype	No. off acc.	Ploidy	Spike	*TtBH*-*A1*																		
				19	134	135	136	137	138	139	140	141	142	143	144	145	146	147	148	209	261	287
CS (Ref.)		6×	Normal	A	C	G	G	C	G	G	C	G	C	G	C	G	G	C	A	A	C	T
tHAP_1	16	4×	Normal	·	·	·	·	·	·	·	·	·	·	·	·	·	·	·	·	·	·	·
tHAP_2	39	4×	Normal	·	·	·	·	·	·	·	·	·	·	·	·	·	·	·	·	·	·	·
tHAP_3	9	4×	Normal	·	·	·	·	·	·	·	·	·	·	·	·	·	·	·	·	·	·	·
tHAP_4	1	4×	smr	·	·	·	·	·	·	·	·	·	·	·	·	·	·	·	·	·	·	·
tHAP_5	1	4×	Normal	·	·	·	·	·	·	·	·	·	·	·	·	·	·	·	·	·	·	·
tHAP_6	49	4×	Branched	·	·	·	·	·	·	·	·	·	·	·	·	·	·	·	·	·	·	C
tHAP_7	1	4×	smr	G	·	·	·	·	·	·	·	·	·	·	·	·	·	·	·	·	·	·

Haplotype	*TtBH*-*B1*											
	37	38	39	40	41	42	43	144	267	491	619	785
CS (Ref.)	T	C	C	C	A	G	A	C	C	A	A	T
tHAP_1	·	·	·	·	·	·	·	·	T	·	A	C
tHAP_2	·	·	·	·	·	·	·	·	·	·	A	T
tHAP_3	·	·	·	·	·	·	·	T	·	G	T	C
tHAP_4	·	·	·	·	·	·	·	·	·	·	A	C
tHAP_5	–	–	–	–	–	–	–	T	·	G	T	C
tHAP_6	·	·	·	·	·	·	·	·	T	·	A	C
tHAP_7	·	·	·	·	·	·	·	·	·	·	A	T

The numbers indicate the nucleotide positions in the corresponding gene. Dots indicate sequences as in the corresponding reference genome. Dashed lines indicate deletion. No. acc, number of accessions; Ref, reference genome cv. Chinese Spring, CS; smr, sham ramification; mrs, multi-row spike

The single accession in tHAP_7, PI 67339 carries a non-synonymous substitution outside of the AP2/ERF region (A19G) of *TtBH*-*A1* and shows the sham ramification (*Shr*) phenotype. Interestingly, the locus, *Shr2*, controlling the sham ramification phenotype from this accession has been mapped on the long arm of chromosome 2A (Amagai et al. 2017). The sham ramification phenotype was also seen in tHAP_4 carrying the functional *TtBH*-*A1* allele. This indicates that the A19G substitution in *TtBH*-*A1* was not the causative mutation for the observed sham ramification phenotype in these two accessions.

### Insertion of ‘Miniature Inverted-repeat Transposable Elements’ (MITE) near *TtBH*-*B1*

**Fig. 8:**
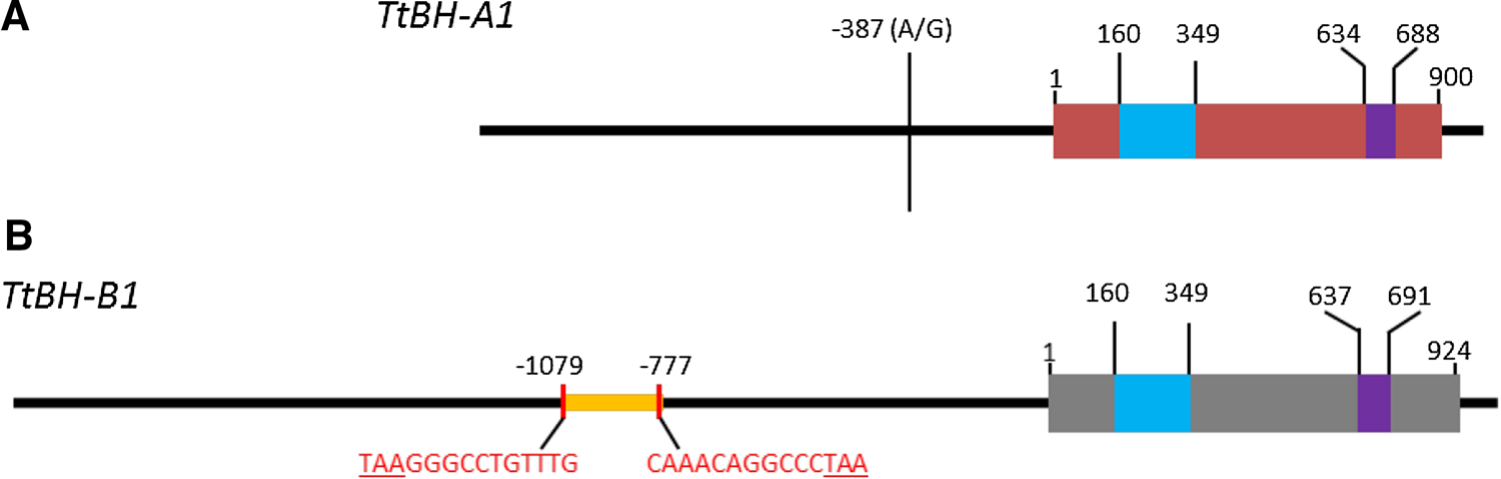
Analysis of the upstream region of *TtBH*-*A1* (1.3 kb) and *TtBH*-*B1* (2.1 kb) using 39 canonical and 28 ‘Miracle wheat’ accessions. No structural variation in the 1.3 kb region of *TtBH*-*A1* was found except for the SNP located at position − 387 from the start codon of the gene (**a**). Only 6 non-spike-branching accessions carry adenine (A), while the remaining 61 accessions, including 28 spike-branching types, carry guanine (G). The MITE insertion (orange) in the vicinity of *TtBH*-*B1* (**b**) was common in all 67 accessions. Sequence for terminal inverted repeats is shown in red with the target site duplication (TAA) underlined. Numbers indicate the position of the nucleotides (color figure online)

To further investigate allelic variation close to the two homoeo-genes, we performed sequence analysis in the putative promoter regions of *TtBH*-*A1* (1.3 kb) and *TtBH*-*B1* (2.1 kb) from 39 canonical and 28 spike-branching accessions. The results are summarized in Fig. 8. For *TtBH*-*A1*, the only sequence variation identified was the SNP located at position -387 (A/G) bp from the start codon (Fig. 8a). From the sequenced accessions (total *n* = 67), only six non-spike-branching accessions carry the adenine (A), while the remaining accessions, including all spike-branching mutants, carry the guanine (G). This result clearly shows that the putative promoter SNP is not linked with the appearance of spike-branching in ‘Miracle wheats.’

For *TtBH*-*B1*, all the sequenced accessions carry the MITE at position − 1079 to − 777 bp (Fig. 8b). Furthermore, we also detected several sequence variations, including insertion and deletions (indels), within *TtBH*-*B1* (Supplementary File 1). Interestingly, the SNP located at position − 54 (G), located in the putative 5′-UTR region (Fig. 3d), was present in all the spike-branching tetraploid accessions (*n* = 49). From 48 canonical accessions, 14 of them also carry the G, suggesting that the SNP is not directly linked with spike-branching phenotype. However, it is important to note that all the 14 accessions carry the functional *TtBH*-*A1* allele that can easily mask the effect of the mutation. We thus hypothesize that the inserted MITE and/or the − 54G close to *TtBH*-*B1* might have contributed to its lowered gene expression in the spike (Fig. 7), or differential tissue expression patterns such as between root and spike. For example, in hexaploid wheat, *TaBH*-*B1* is also expressed in roots where in some cases the expression exceeds that of *TaBH*-*A1* (Supplementary Figure 3).

### Alleles at the *Q* locus do not directly contribute to spike-branching in tetraploid wheat

Almost all ‘Miracle wheats’ carry the *q*^*del*^-*5A* allele, making them double mutants for the two important genes involved in the SM identity (*bh*^*t*^-*A1*) and SM determinacy (*q*^*del*^-*5A)* (Poursarebani et al. 2015; Wolde et al. 2019b). Since the Q protein is involved in spike development through rachis and rachilla morphogenesis, we hypothesized that the *q*^*del*^-*5A* allele might also be involved in spike-branching. We therefore re-mapped spike-branching after phenotyping the same mapping population (i.e., F9 RILs) grown in the greenhouse. This time, phenotyping was performed by using a qualitative scale from 0 (non-branching) to 3 (spike-branching with mini-spike branches), thereby trying to quantify genuine spike-branching instead of additional spikelet per spike alone. However, spike-branching was not mapped to the region harboring *q*^*del*^-*5A* allele.

**Fig. 9:**
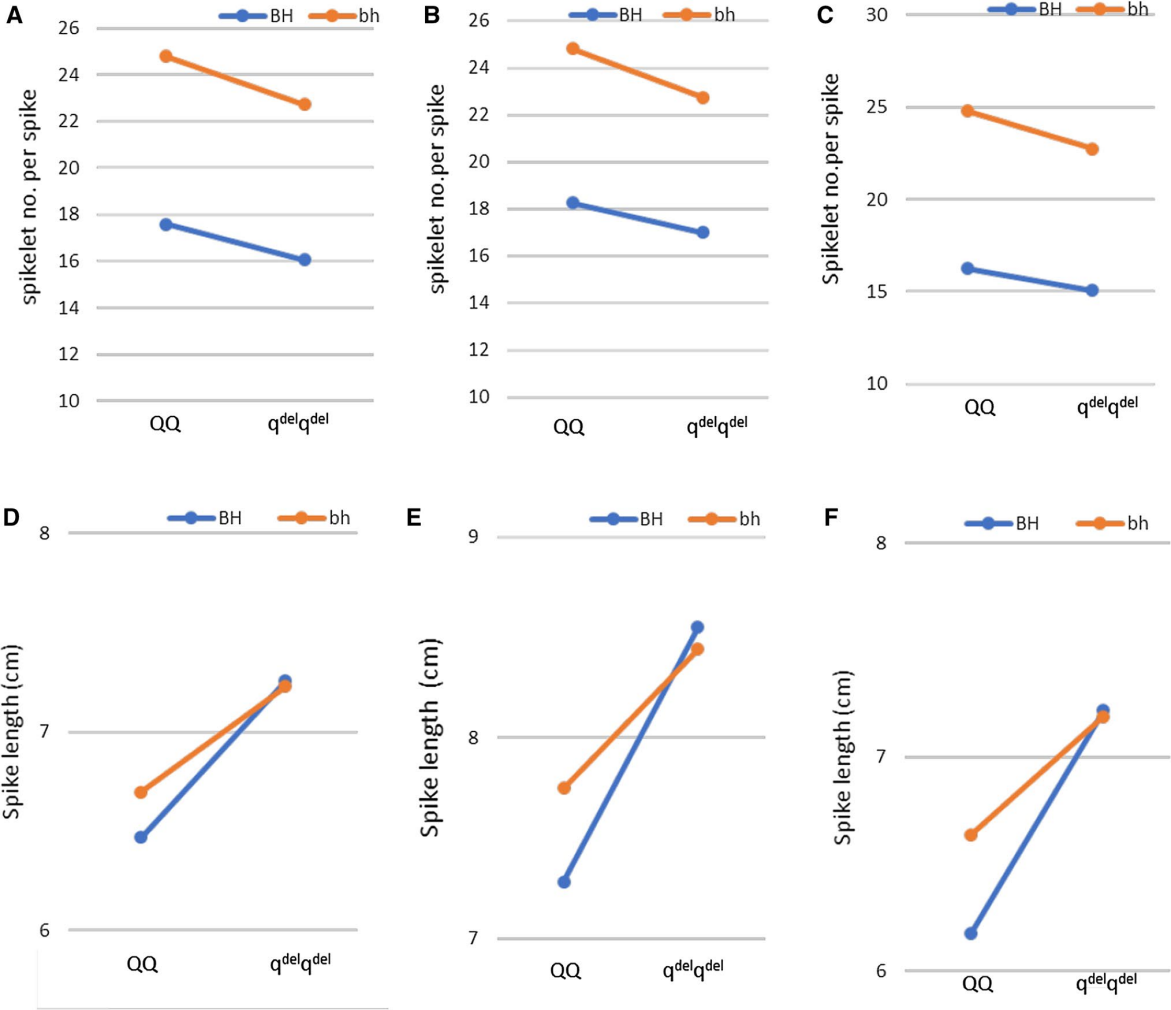
Epistatic interaction effects between *bh*^*t*^-*A1* and *q*^*del*^-*5A* are depicted as reaction norms. Values shown are the mean trait value (*y*-axis) from three different environments (**a**, **d** IPK14; **b**, **e** IPK15; and **c**, **f** HAL15). Group of homozygous genotypes, grouped based on gene-derived CAPS markers, are shown on the *x*-axis (blue line represents the reaction norm of RILs carrying the *TtBH*-*A1* wild-type allele, BH, on 2AS; while RILs with the mutant allele, bh, are shown in orange). Epistasis is indicated by the differences in slopes of the lines connecting each group. Since the lines are parallel, i.e., equal slope, no epistatic interaction was detected between *bh*^*t*^-*A1* and *q*^*del*^-*5A* alleles for the spikelet number per spike in all the three environments (**a** IPK14; **b** IPK15; and **c** HAL15), while a clearer interaction between *bh*^*t*^-*A1* and *q*^*del*^-*5A* affecting spike length in all the three environments could be found (**d** IPK14; **e** IPK15; and **f** HAL15) (color figure online)

Notably, previous studies also failed to map spike-branching to this chromosomal arm (Dobrovolskaya et al. 2009; Echeverry-Solarte et al. 2014), indicating that the Q protein might not directly contribute to spike-branching. However, consistent with previous studies (Simons et al. 2006), spike or rachis length was mapped to chromosome 5AL at the *q* locus, thereby linking spike morphometric traits to the *q*^*del*^-*5A* allele (Wolde et al. 2019b). We further looked at whether an epistatic interaction exists between *bh*^*t*^-*A1* and *q*^*del*^-*5A.* Consistent with our mapping result, we did not detect an epistatic interaction between *bh*^*t*^-*A1* and *q*^*del*^-*5A* for spikelet number per spike (Fig. 9a–c). This was not surprising as Q does not affect spikelet number but rather floret number per spikelet via rachilla elongation (Debernardi et al. 2017; Greenwood et al. 2017). Interestingly, we detected an epistatic interaction between *bh*^*t*^-*A1* and *q*^*del*^-*5A* for the spike (rachis) length (Fig. 9d–f). This might suggest an indirect effect of the *q*^*del*^-*5A* allele on the morphogenesis of the ‘mini-spikes’-like branches of ‘Miracle wheat’ regardless of the spikelet number on the ‘mini-spikes.’

## Discussion

### Identification of loci modifying spike-branching in tetraploid wheat

Previously, we identified the recessive SM identity gene, *TtBH*-*A1,* from 2AS as a suppressor of spike-branching in tetraploid wheat (Poursarebani et al. 2015). However, the exact role of the homoeo-gene from 2BS, *TtBH*-*B1*, remained unknown. Among the two newly detected QTL identified in this study (1AS and 2BS), *QSS.ipk*-*2BS* is highly associated with allelic variation at *TtBH*-*B1* (Fig. 3e). The complete association of the *TtBH*-*B1* haplotype (coding T785C/L262S substitution) with the occurrence of the ‘Miracle wheat’ phenotype points to the presence of an allele with lowered functionality. This was evident from RILs carrying both alleles from TRI 19165 showing increased penetrance of SS and/or mini-spike formation as compared with RILs carrying only one of the recessive homoeo-alleles (aaBB) (Fig. 4). Furthermore, the enhanced penetrance and expressivity of SS/mini-spike formation in most of the ‘Miracle wheat’ accessions suggests a reduced or modifying role of *TtBH*-*B1* during spike development.

In hexaploid wheat, the multi-row spike (MRS) is controlled by the homoeo-alleles *WFZP*-*D1* and *WFZP*-*A1* (Dobrovolskaya et al. 2015; Echeverry-Solarte et al. 2014). The combined effect from *WFZP*-*A1* and *WFZP*-*D1* has been reported as the two major effect alleles (Dobrovolskaya et al. 2009, 2015). Our results from tetraploid wheat show that *TtBH*-*A1* is indeed the major effect gene followed by *TtBH*-*B1* (Fig. 3a). Dobrovolskaya et al. (2015) reported that *WFZP*-*B1* was not expressed due to the MITE insertion in the promoter region (Dobrovolskaya et al. 2015). But our results clearly show that despite the inserted MITE close to *TtBH*-*B1*, the homoeo-gene is still expressed in developing spike tissues in tetraploid wheat (Fig. 7).

We also looked at the transcript expression pattern of *TtBH*-*A1* and *TtBH*-*B1* in the hexaploid Wheat Gene Expression Atlas (Ramirez-Gonzalez et al. 2018). Results showed that both genes are well expressed in root tissues, where in some cases, *TtBH*-*B1* is even more expressed than *TtBH*-*A1* at different stages of the root development. This rather suggests that *TtBH*-*A1* and *TtBH*-*B1* are differentially tissue-regulated (Supplementary Figure 3).

Different studies have also shown that MITEs are favorably inserted in the vicinity of genes and play a critical role in gene regulation and genome evolution through promoter enhancement and repression including epigenetic modification of the promoter (Hou et al. 2012; Naito et al. 2006, 2009; Yaakov et al. 2013; Yang et al. 2005). Since the MITE is detected within 2.3 kb upstream of *TtBH*-*B1* (Fig. 8b), it might have a similar role in the modification of the promoter and thus gene regulation. Therefore, further studies are necessary for more insights into the effect of the MITE in the regulation of *TtBH*-*B1/WFZP*-*B1*, especially for a better understanding of epigenetic factors controlling the phenotypic penetrance and expressivity of spike-branching in wheat.

So taken together, based on (1) the tight linkage between the *TtBH*-*B1*-derived CAPS marker and *QSS.ipk*-*2BS,* (2) the found differences in the mRNA expression levels of the two homoeo-genes (i.e., *TtBH*-*A1* and *TtBH*-*B1*), (3) the independent confirmation from the biparental QTL mapping and through GWAS analyses, and (4) the association of non-synonymous substitutions of *TtBH*-*B1* with spike-branching (haplotype analysis; L262S substitution), it is very likely that *QSS.ipk*-*2BS* is the *bh*^*t*^-*B1* allele of *TtBH*-*B1*. However, further causative follow-up analyses are still required to fully elucidate the causative nature of this allele and its contribution to spike and root architecture in tetraploid wheat.

### Spike architecture and phenotypic plasticity in spike-branching wheat mutants

The architecture of the grass inflorescence is determined by the fate of the inflorescence meristem (IM). Branching of the inflorescence occurs when the IM produces a branch meristem (BM). Since the BM formation in wheat is highly suppressed, the IM directly produces the SM. Therefore, the wheat spike becomes determinate by forming a terminal spikelet (Koppolu and Schnurbusch 2019; Sakuma and Schnurbusch 2020). On the contrary, the spike of ‘Miracle wheat’ rather remains indeterminate (often without terminal spikelet) thereby producing lateral meristems that produce mini-spike-like branches predominantly from the basal part of the main axis of the spike; suggesting loss of SM identity and determinacy (Poursarebani et al. 2015). Interestingly, the mini-spike-like branches can produce their own spikelets in a distichous arrangement resulting in an indeterminate number of spikelets per spike. Even though such mini-spike-like branches are typical characteristics of spike-branching in wheat, our near-isogenic lines of *QSS.ipk*-*2AS* (*bh*^*t*^-*A1*) only show SS formation (Wolde et al. 2019a), clearly indicating yet unidentified branch-suppressing gene(s) in the wheat genome. Indeed, spike-branching is determined not only by the genetic composition of the genotype but also by environmental factors (Pennell and Halloran 1984a, b; Sharman 1944). Hence, the phenotypic plasticity of SS formation across different environments and the estimated genetic coefficient of variation (GCV) and narrow-sense heritability (*h*^2^) are shown in Supplementary Table 2.

To further test the phenotypic plasticity of spikelet number in the mutant RILs, we compared SS formation from RILs showing spike-branching and/or SS formation from spike samples deliberately collected from the border and middle rows of each plot. This is based on the premise that border plants have relatively better access to resources, such as light, water and nutrients, as compared to the more crowded plants in the middle rows of the plots. Unsurprisingly, samples from the borders had a significantly higher number of SS as compared to their counterparts from the middle rows of the plot which signifies the effect of even a microenvironment on the phenotypic plasticity of spike-branching (Supplementary Figure 4). However, QTL mapping using the data obtained from the border- and middle-row plants did not result in new QTL other than *QSS.ipk*-*2AS* and *QSS.ipk*-*2BS*, implying that the observed phenotypic variation in spikelet number is indeed due to phenotypic plasticity in response to environmental factors. Taken together, these results show that the loss of SM identity/determinacy in ‘Miracle wheats’ combined with environmental factors control the phenotypic plasticity of SS in the RILs.

Besides the exhibited phenotypic plasticity in ‘Miracle wheat’, branch and/or SS formation in ‘Miracle wheat’ or ‘Miracle wheat’-derived lines are sometimes absent even among plants/tillers of the same genotype and produce a non-branching plant. Nevertheless, the mechanistic basis of such irregularities in the phenotypic expression of spike-branching is still unknown. Although epigenetic mechanisms have been proposed as key factors in plant phenotypic plasticity, the role of epigenetics in the phenotypic plasticity of spike-branching remains unclear.

### Effects of SS formation and/or spike-branching on spikelet fertility

Although ‘Miracle wheat’ generally has a higher grain number per spike due to the increased spikelet number, fertility of the individual spikelet is very low, often only around one grain per spikelet (Supplementary Figure 5). This might have been one likely reason for breeders to deselect ‘Miracle wheat’. In fact, *bh*^*t*^-*A1* and *bh*^*t*^-*B1* increase the sink size, but the lowered spikelet fertility signifies the trade-off with spike-branching (Supplementary Figure 6).

Our results show that about 27.6% of the observed phenotypic variance for lowered spikelet fertility has been linked to *QSS.ipk*-*2AS* or *bh*^*t*^-*A1*, which is followed by *QSS.ipk*-*2BS* or *bh*^*t*^-*B1* (about 11.5%) (Supplementary Table 5). Two possible reasons for the trade-off between spike-branching and spikelet fertility are very important. First, it might be related to the loss of the SM identity/determinacy that might result in unsynchronized development of SS and/or floret on the spike. Secondly, the trade-off between spike-branching and spikelet fertility is due, partly, to competitional effects between spikelets and/or florets for resources, which of course needs proof.

### Increasing sink size is insufficient for increasing wheat yield without increasing spikelet fertility

Wheat yield increment due to systematic improvement in the Harvest Index (HI), i.e., partitioning of above-ground dry matter (DM) to grain yield, is currently being stagnating; signaling the hypothetical limit of the partitioning of DM to grain yield might have reached the ceiling (Austin et al. 1980; Foulkes et al. 2011; Ray et al. 2012). This hypothesis is also clearly manifested in our study by the negative association between sink size (spikelet number) with spikelet fertility (Fig. 6b, e). This indicates that increasing sink size alone is not sufficient for increasing wheat yield without improving floret or spikelet fertility (Sakuma et al. 2019; Sakuma and Schnurbusch 2020). Nevertheless, the increased spikelet number still resulted in more grains per spike and interestingly, without significantly affecting grain weight, i.e., TKW (Fig. 6d, f). In fact, several endogenous and environmental factors affect grain weight; and thus, the negative correlation between grain number and TKW does not necessarily reflect the effect of competition among grains (Foulkes et al. 2011; Golan et al. 2019; Quintero et al. 2018; Slafer 2007). Different studies have also indicated that besides increasing grain yield, the grains from semi-dwarf wheat varieties are also smaller in size (Casebow et al. 2016; Jobson et al. 2019). Because semi-dwarf wheat varieties are resistant to lodging and more responsive to a higher dose of fertilizer (Flintham et al. 1997; Jobson et al. 2019), more than 70% of commercial wheat cultivars worldwide have the semi-dwarfing genes (Hedden 2003). In line with this, *Rht*-*B1* is the most widely used allele to ensure short plant stature in tetraploid wheat. Therefore, by moderately increasing spikelet numbers, there still be a possibility of increasing wheat yield without negatively affecting grain weight (Wolde et al. 2019a).

## Conclusion

Identifying genes controlling meristem identity and determinacy are important to understand the genetic and molecular basis of wheat spike development. So far, little is known about meristem identity and determinacy genes and their networks in wheat. Besides the previously identified SM identity gene, *TtBH*-*A1*, we have identified new ‘Miracle wheat’ loci on chromosome 1AS and 2BS that have a significant effect on modifying the expressivity of spike-branching in tetraploid wheat.

## Electronic supplementary material

Below is the link to the electronic supplementary material.

Supplementary material 1 (XLSX 72 kb)

Supplementary material 2 (DOCX 680 kb)
