# Supplementary material for: Genome-wide identification of loci modifying spike-branching in tetraploid wheat
Source: Theor Appl Genet. 2021 May 7;134(7):1925–43. doi: 10.1007/s00122-020-03743-5 (PMC8263435; doi:10.1007/s00122-020-03743-5)
Supplement: Supplementary file 2 — Supplementary material 2 (DOCX 680 kb) [file 122_2020_3743_MOESM2_ESM.docx]

**Supplementary Tables and Figures**

**Supplementary Table 1.** Comparison of the parent, mutant checks, and RILs for additional and total spikelets per spike across four different environments.

| Trait | Environment | Parent |  | Mutant Check |  | RILs(n=146) | | | |
| --- | --- | --- | --- | --- | --- | --- | --- | --- | --- |
|  |  | Bellaroi |  | (n=3) |  | Min | Max | Mean | SD |
| Additional Spikelet/Spike | IPK14 | - |  | 32.92 |  | 0.00 | 34.15 | 7.51 | 5.66 |
| (addSPS) | IPK15 | - |  | 50.83 |  | 0.00 | 35.12 | 10.40 | 7.74 |
|  | HAL15 | - |  | 35.15 |  | 0.00 | 21.58 | 6.39 | 4.07 |
|  | GH15 | - |  | 45.31 |  | 0.00 | 14.50 | 3.04 | 4.05 |
| Total Spikelet/Spike | IPK14 | 13.75 |  | 52.37 |  | 12.60 | 51.35 | 21.02 | 6.06 |
| (totSPS) | IPK15 | 14.90 |  | 71.57 |  | 12.20 | 54.52 | 23.82 | 8.41 |
|  | HAL15 | 12.74 |  | 53.78 |  | 11.48 | 39.46 | 19.78 | 5.36 |
|  | GH15 | 12.75 |  | 69.44 |  | 12.00 | 34.00 | 20.13 | 4.89 |

SD, Standard Deviation

**Supplementary Table 2**. Mean Squares, Genotypic Coefficient of Variation (GCV), and narrow-sense heritability (h^2^) of additional spikelets per spike (addSPS) and total spikelets per spike (totSPS). Data were combined from three different environments: IPK14, IPK15, HAL15. GEI, Genotype-by- Environment Interaction.

| Trait | Genotype | Environment | GEI | Residual | GCV (%) | h^2^(%) |
| --- | --- | --- | --- | --- | --- | --- |
| addSPS | 183.91 ** | 401.26 ** | 13.38 ** | 4.234 | 138.47 | 76 |
| totSPS | 239.4 ** | 1237.48 ** | 15.28 ** | 5.64 | 28.45 | 78 |

**Supplementary Table 3**. Quantitative trait loci linked with supernumerary-spikelet in the RILs.

| QTL | Position(cM) | −log10 (P-value) | Environment | PVE (%) | QTL Additive Effect | HVA |
| --- | --- | --- | --- | --- | --- | --- |
| *QSS.ipk-1AS* | 3.9 | 4 - 5 | IPK15, HAL15 | 5 - 6 | 1.01 - 1.71 | TRI 19165 |
| *QSS.ipk-2AS* | 39.1 | 13 - 20 | GH15, IPK14, HAL15, IPK15 | 27 - 32 | 2.17 - 4.11 | TRI 19165 |
| *QSS.ipk-2BS* | 52.4 | 11 - 17 | GH15, IPK14, HAL15, IPK15 | 22 - 26 | 1.64 - 3.54 | TRI 19165 |

PVE, Phenotypic Variance Explained; HVA, High-Value Allele

**Supplementary Table 4.** Epistatic interactions and estimation of the additive-by-additive effect of SS QTL.

A positive value indicates that the parental (TRI 19165) two-locus genotypes have a positive effect and the recombinants had a negative effect. Although no QTL has been mapped on chromosome 5B, the specified interval was found to interact with *QSS.ipk-1AS* and *QSS.ipk-2AS.*

| QTL Interaction |  | Additive by Additive (AA) effect |  | Heritability |
| --- | --- | --- | --- | --- |
| *QSS.ipk-1AS X QSS.ipk-2BS* |  | 0.817 |  | 0.018 |
| *QSS.ipk-1AS X 5BS** |  | -0.522 |  | 0.005 |
| *QSS.ipk-2AS X QSS.ipk-2BS* |  | 1.66 |  | 0.062 |
| *QSS.ipk-2AS X 5BS** |  | -0.606 |  | 0.003 |

5BS* Shows the interval on chromosome 5BS between marker 5BS_2235368:6154 and 5BS_2292762:2732.

**Supplementary Table 5.** Effect of the *QSS.ipk-2A* and *QSS.ipk-2B* on grain number per spikelet (GPS)

| QTL |  | Pos.(cM) |  | −log10 (P-value) |  | Trait Affected |  | Environment |  | Effect |  |  | PVE (%) | HVA |
| --- | --- | --- | --- | --- | --- | --- | --- | --- | --- | --- | --- | --- | --- | --- |
| *QSS.ipk-2AS* |  | 39.1 |  | 16 - 34 |  | GPS |  | IPK14, IPK15, HAL15 |  | 0.41 - 0.53 |  |  | 16.9 - 27.6 | Bellaroi |
| *QSS.ipk-2BS* |  | 52.4 |  | 6 - 17 |  | GPS |  | IPK14, IPK15, HAL15 |  | 0.24 - 0.34 |  |  | 5.9 - 11.5 | Bellaroi |

**
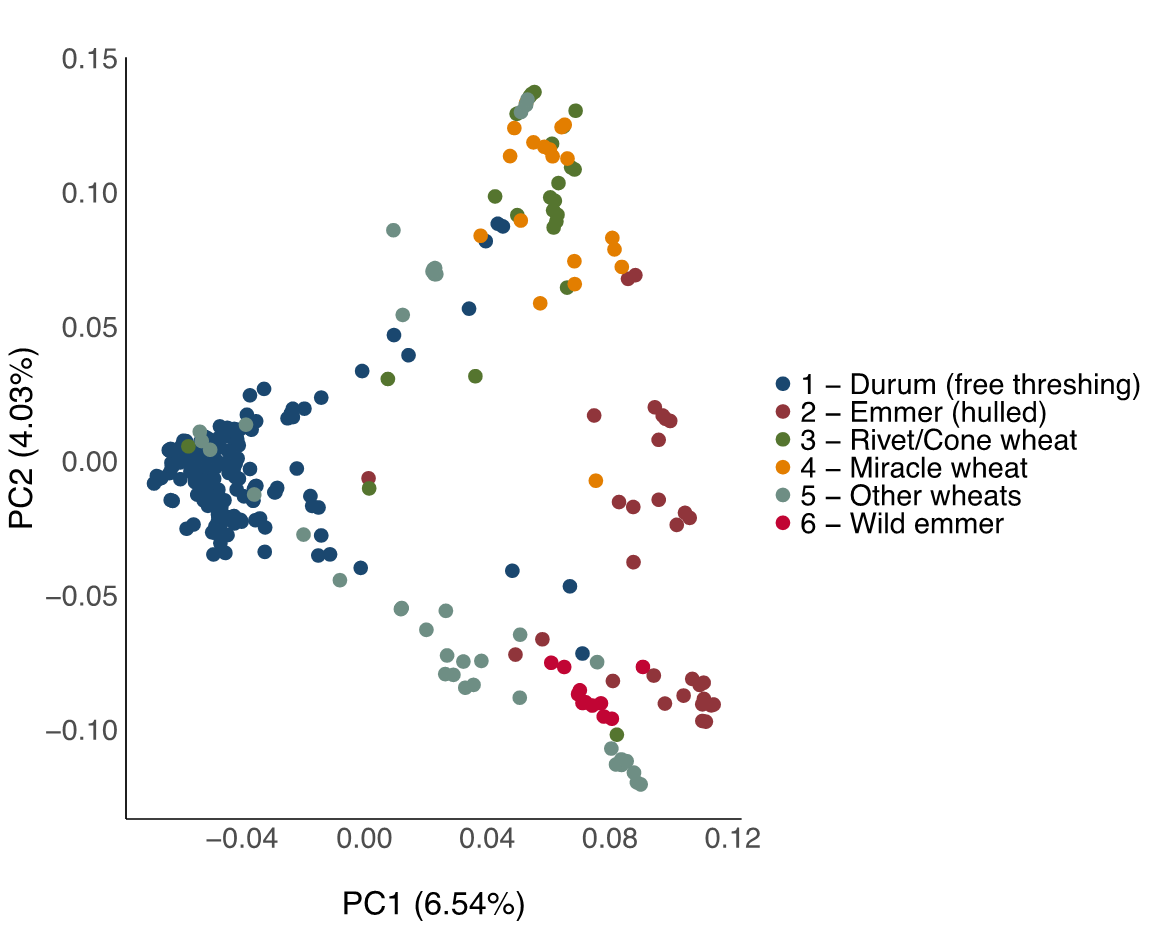
**

**Supplementary Figure 1.** Principal Component Analysis of accessions used for the GWAS analysis


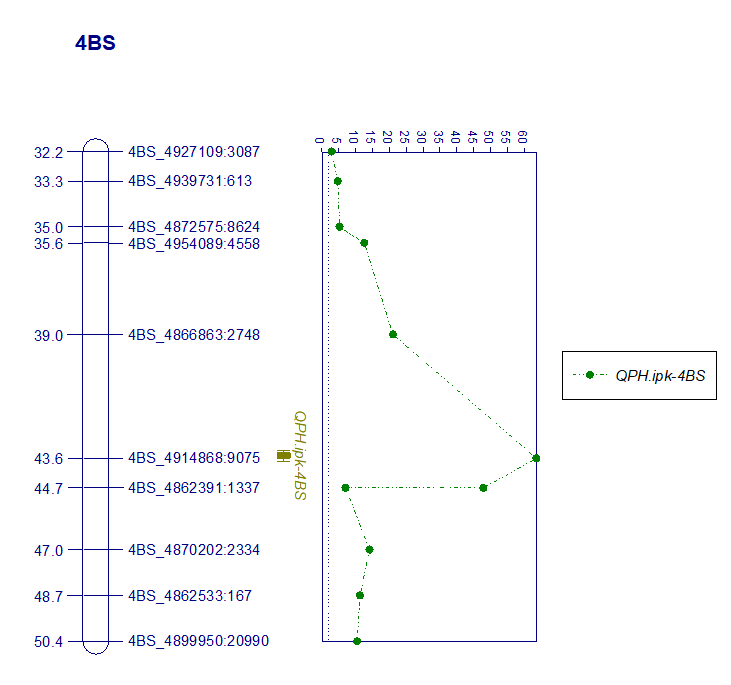


**Supplementary Figure 2.** Plant height QTL mapped to chromosome 4BS


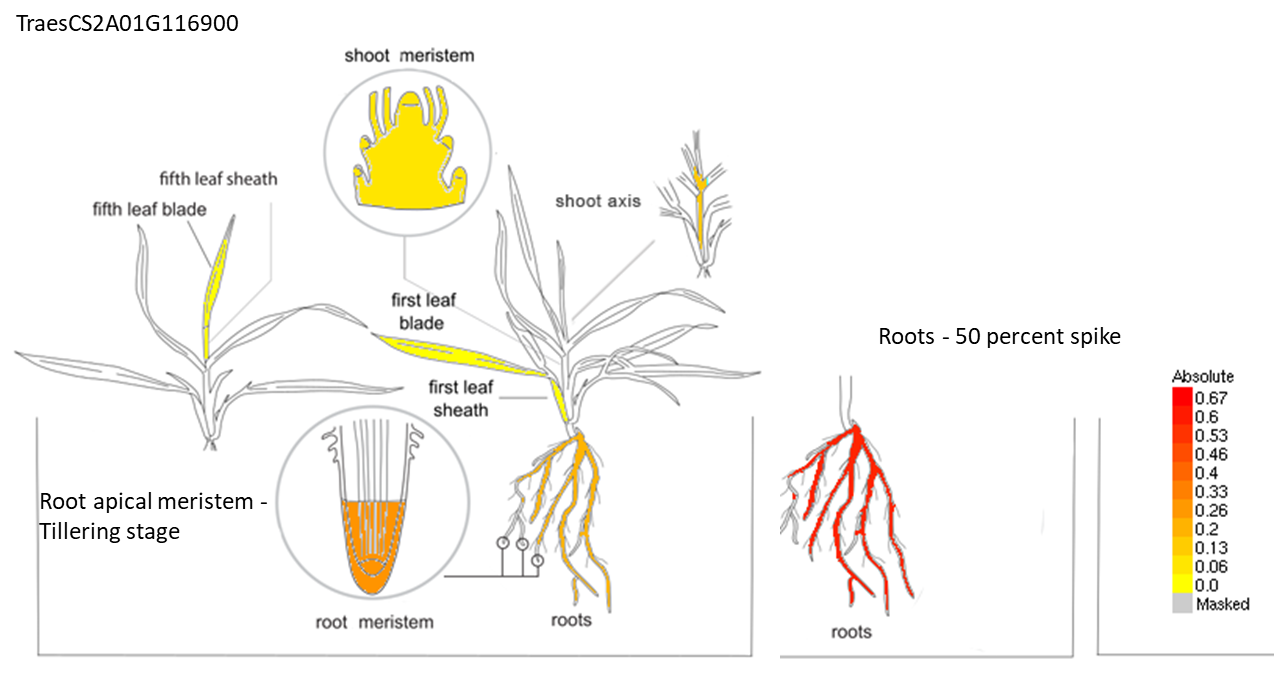


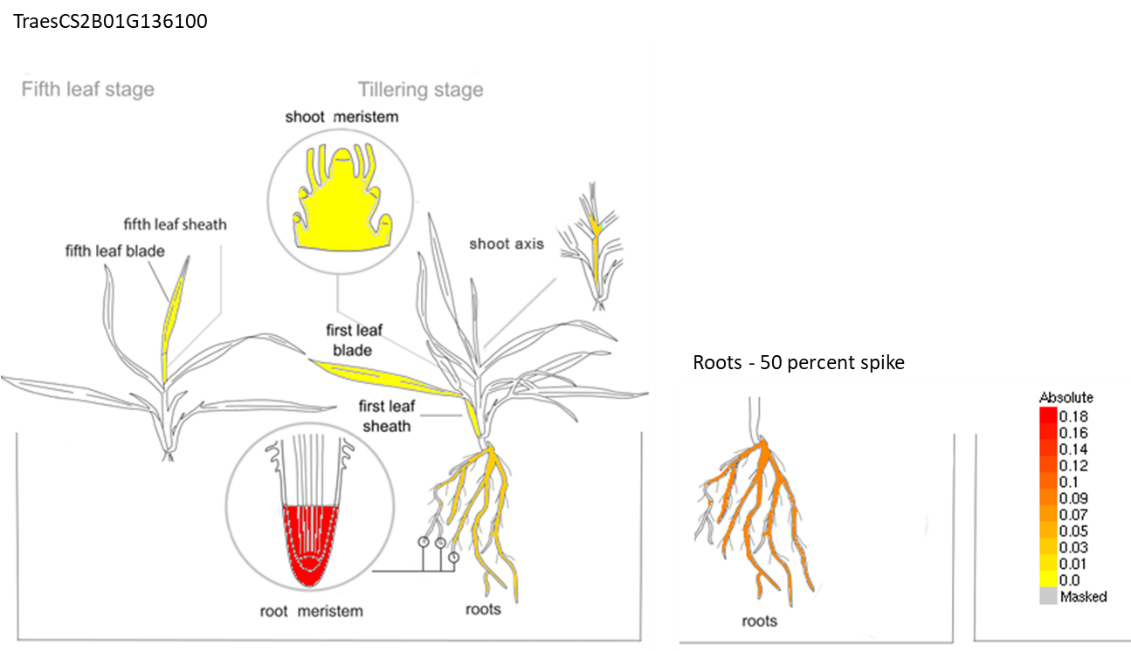


**Supplementary Figure 3.** Expression of *TaBH-A1* (TraesCS2A02G116900), *TaBH-B1* (TraesCS2B02G136100) at different stages of roots development. Analysis was made using publicly available wheat eFP browser ( <https://bar.utoronto.ca/efp_wheat/cgi-bin/efpWeb.cgi>)


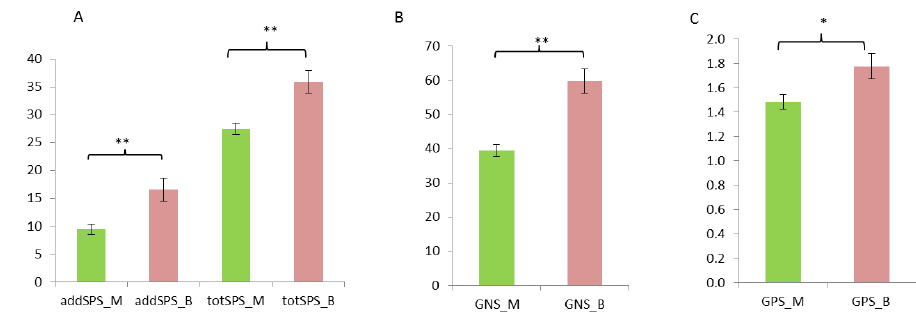


**Supplementary Figure 4.** Phenotypic plasticity of SS formation.

The comparison was made from field-grown plants collected from the border and middle row of each plot. addSPS, totSPS, GPS, and GNS were derived from medium to strongly branching RILs (genotype: *aabb;* n=39) from field grown RILs in 2014 (IPK14). (A) Additional and total spikelet per spike, (B) Grain number per spike, (C) Grain number per spikelet. A significance level was calculated based on the unpaired two-tailed *student’s t-test* at *P*= 0.05. addSPS, additional spikelet per spike; totSPS, total spikelet per spike; GNS, grain number per spike; GPS, Grain number per spikelet M, Middle; B, Border.

**Supplementary Figure 5.** Comparison between standard wheat spike and ‘Miracle wheat’ for grain number per spike and spikelet. Bel, Bellaroi; SBMs, Spike-branching mutants (TRI 9652, TRI 3261, and TRI 5283); GNS, grain number per spike.


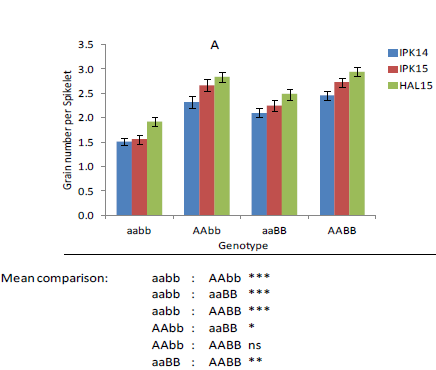


**Supplementary Figure 6.** Comparison of groups of RILs for spikelet fertility. RILs that have combined both *QSS.ipk-2A* and *QSS.ipk-2B* (=aabb; n=39) show strong spike-branching and or/SS formation but have low grain numbers per spikelet. RILs that only have *QSS.ipk-2A* (=aaBB; n=30) show weak spike-branching or less SS formation but higher grain number per spikelet. Error bars are mean ± SEM. The mean comparison was made using unpaired *Student’*s two-tailed *t-test* at *p*-value 0.05, *; 0.01, **; and 0.001, ***. ns, non-significant.
